# Lessons From Insect Fungiculture: From Microbial Ecology to Plastics Degradation

**DOI:** 10.3389/fmicb.2022.812143

**Published:** 2022-05-24

**Authors:** Mariana O. Barcoto, Andre Rodrigues

**Affiliations:** ^1^Center for the Study of Social Insects, São Paulo State University (UNESP), Rio Claro, Brazil; ^2^Department of General and Applied Biology, São Paulo State University (UNESP), Rio Claro, Brazil

**Keywords:** microbiota, xenobiotics, bioremediation, pollutants, plant, polymers, lignocellulose, symbiosis

## Abstract

Anthropogenic activities have extensively transformed the biosphere by extracting and disposing of resources, crossing boundaries of planetary threat while causing a global crisis of waste overload. Despite fundamental differences regarding structure and recalcitrance, lignocellulose and plastic polymers share physical-chemical properties to some extent, that include carbon skeletons with similar chemical bonds, hydrophobic properties, amorphous and crystalline regions. Microbial strategies for metabolizing recalcitrant polymers have been selected and optimized through evolution, thus understanding natural processes for lignocellulose modification could aid the challenge of dealing with the recalcitrant human-made polymers spread worldwide. We propose to look for inspiration in the charismatic fungal-growing insects to understand multipartite degradation of plant polymers. Independently evolved in diverse insect lineages, fungiculture embraces passive or active fungal cultivation for food, protection, and structural purposes. We consider there is much to learn from these symbioses, in special from the community-level degradation of recalcitrant biomass and defensive metabolites. Microbial plant-degrading systems at the core of insect fungicultures could be promising candidates for degrading synthetic plastics. Here, we first compare the degradation of lignocellulose and plastic polymers, with emphasis in the overlapping microbial players and enzymatic activities between these processes. Second, we review the literature on diverse insect fungiculture systems, focusing on features that, while supporting insects’ ecology and evolution, could also be applied in biotechnological processes. Third, taking lessons from these microbial communities, we suggest multidisciplinary strategies to identify microbial degraders, degrading enzymes and pathways, as well as microbial interactions and interdependencies. Spanning from multiomics to spectroscopy, microscopy, stable isotopes probing, enrichment microcosmos, and synthetic communities, these strategies would allow for a systemic understanding of the fungiculture ecology, driving to application possibilities. Detailing how the metabolic landscape is entangled to achieve ecological success could inspire sustainable efforts for mitigating the current environmental crisis.

## Introduction

A mark of human evolution, the adaptability to novel resources and environments led to drastic human-caused changes in land surface, atmosphere, oceans, landscapes structure, climate, weather patterns, and biogeochemical cycles (Tilman and Lehman, 2001; Lewis and Maslin, 2015; Keys et al., 2019). Anthropogenic activities have transformed about 30–50% of the biosphere composition (Bar-On et al., 2018; Chure et al., 2021), ultimately reorganizing life on Earth (Lewis and Maslin, 2015; Keys et al., 2019). With the anthropogenic mass outnumbering all living biomass (Elhacham et al., 2020), global pollution is one of the Anthropocene hallmarks (Porta, 2021). Human-made compounds are synthesized for industrial, agricultural, and domestic applications, gathered under the term “xenobiotic” that embrace plastics, polycyclic aromatic hydrocarbons (PAHs), pharmaceutical active compounds, and pesticides (Embrandiri et al., 2016; Atashgahi et al., 2018; Mishra et al., 2021). On one hand, accumulating agroindustrial bio-waste and xenobiotic pollutants are crossing boundaries of planetary threat while causing a global crisis of waste overload (Rockström et al., 2009; Persson et al., 2013; de Lorenzo et al., 2016; Chure et al., 2021). On another hand, some of these recalcitrant waste materials are potential sources of energy and value-added products to be explored through the wide metabolic diversity of microorganisms (Rittmann et al., 2008; Tuck et al., 2012; Wigginton et al., 2012; Pagliano et al., 2017; Lag-Brotons et al., 2020; Ozbayram et al., 2020).

**FIGURE 1:**
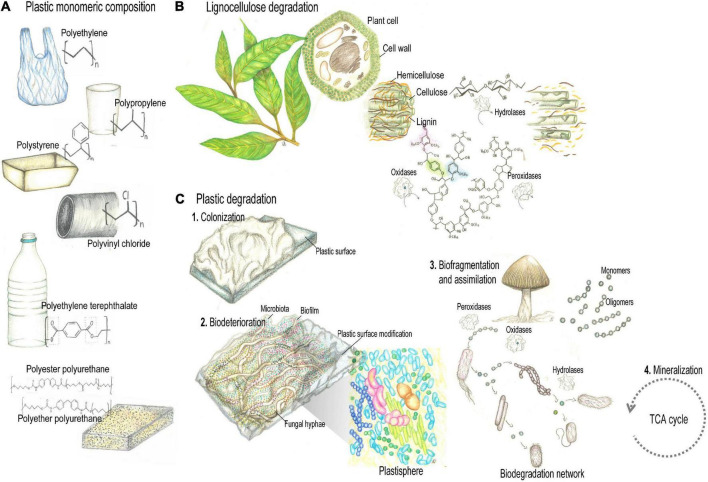
Plant and synthetic polymers degradation **(A)**. Petroleum-derived polyethylene (PE), polypropylene (PP), polystyrene (PS), polyvinylchloride (PVC), polyurethanes (PUR), and polyethylene terephthalate (PET) comprise the majority of plastic polymers currently produced. **(B)** Despite fundamental differences regarding structure and recalcitrance, lignocellulose and plastic polymers share physical-chemical properties to some extent, that include carbon skeletons with similar chemical bonds, hydrophobic properties, amorphous and crystalline regions. Thus, microbial strategies to deal with such properties would allow enzymes with lignocellulolytic activity to depolymerize plastics. **(C)** Plastic biodegradation encompasses processes of bio-deterioration and bio-fragmentation. Enzymes active during the biodegradation of synthetic plastic polymers include hydrolases and oxidoreductases. Pencil drawing illustrations by Mariana Barcoto.

Plastic-degrading capacity has been observed in bacterial and fungal species sampled from diverse polluted environments (Montazer et al., 2020), such as: waste soil (Orr et al., 2004; Mor and Sivan, 2008); oil and petroleum-contaminated soil (Jeon and Kim, 2014, 2015); compost (Yoshida et al., 2016); solid waste and plastic debris (Hadad et al., 2005; Usha et al., 2011; Das and Kumar, 2015; Peixoto et al., 2017); waste water and activated sludge (Wei et al., 2020); shallow and pelagic sea water (Sudhakar et al., 2008; Harshvardhan and Jha, 2013; Kumar et al., 2021). Microbial enzymatic activity related to plastic polymers degradation includes oxidoreductases (as laccases, peroxidases, lytic polysaccharide monoxygenases), and hydrolases (as cutinases, amidases, peptidases, and lipases; Daly et al., 2021). In nature, microbial oxidoreductases and hydrolases complimentary degrade recalcitrant components of plant cell walls, the most abundant organic carbon reservoir on Earth (Kirk and Farrell, 1987; Pauly and Keegstra, 2008; Ruiz-Dueñas and Martínez, 2009; Gilbert, 2010; Zhao et al., 2012; Daly et al., 2021). Plant cell walls are composed mainly by lignocellulose, an intricated mesh of cellulose, hemicelluloses, and lignin (Figure 1A; Pauly and Keegstra, 2008; Zhao et al., 2012). Molecular assciations between these components render recalcitrant lignocellulosic fibers, imposing physical-chemical barriers for biodegradation (Malherbe and Cloete, 2002; Zhao et al., 2012). Lignocellulolytic activity starts with an oxidative attack to depolymerize lignin, the most recalcitrant cell wall component, which allow hydrolases to access complex polysaccharides, as cellulose and hemicelluloses (Kirk and Farrell, 1987; Ruiz-Dueñas and Martínez, 2009; Gilbert, 2010).

Despite fundamental differences regarding structure and recalcitrance, lignocellulose and plastic polymers share physical-chemical properties to some extent, that include carbon skeletons with similar chemical bonds (Daly et al., 2021), hydrophobic properties (Notley and Norgren, 2010), amorphous and crystalline regions (Park et al., 2010; Wei and Zimmermann, 2017). Thus, microbial strategies to deal with such properties would allow enzymes with lignocellulolytic activity to depolymerize plastics (Figure 1; Daly et al., 2021). Indeed, lignin-modifying oxidoreductases act by non-specific radical based oxidation, targeting not only the chemical bonds and phenolic subunits of lignin, but also those of plastics, aromatic hydrocarbons, chlorophenols, and aromatic dyes (Mester and Tien, 2000; Daly et al., 2021; Kavitha and Bhuvaneswari, 2021; Zhuo and Fan, 2021). For these features, lignocellulolytic enzymes are regarded as promising candidates for bioremediation of environmental pollutants, comprising a more effective and ecofriendly alternative (Zhuo and Fan, 2021). Ecological strategies for metabolizing recalcitrant polymers have been selected and optimized through evolution. Understanding natural processes for lignocellulose modification could aid the challenge of dealing with the recalcitrant human-made chemicals and polymers spread worldwide (de Lorenzo et al., 2016; Timmis et al., 2019; Daly et al., 2021).

Bioremediation biotechnology could look for inspiration in the diverse and efficient metabolic approaches to degrade, modify, and utilize recalcitrant lignocellulosic materials evolved throughout the tree of life (Cragg et al., 2015; Andlar et al., 2018; Gambarini et al., 2021). Degrading plant biomass in nature often occur at a community level, integrating microbial enzymatic cocktails to synergistically degrade the lignocellulose components (Purahong et al., 2016; Jiménez et al., 2017; Rosnow et al., 2017; Alessi et al., 2018; Bredon et al., 2020). Only in association with lignocellulose-degrading microbial communities, animal hosts can derive nutrients and energy from recalcitrant biomass otherwise poorly digestible (Troyer, 1984; Hansen and Moran, 2014; Hardy et al., 2020). Microbial symbionts paved the way for the rise of herbivory, considered to be a major evolutionary transition leading to phenotypic and behavioral plasticity, and niche construction (Gilbert, 2020). Herbivorous hosts access plant nutrients in a “holobiont level”, i.e., by physiological processes of the host and its associated microbiota; Bredon et al., 2018; Simon et al., 2019; Gilbert, 2020; Moeller and Sanders, 2020). Charismatic examples of host-microorganism associations for exploring plant-derived niches are found in fungus-cultivating insects. Fungiculture embraces passive or active fungal cultivation, where the insect takes advantage of fungal plant-decomposing capacity for nourishment and/or protection. Fungi, in turn, take advantage of maintenance and propagation. These symbioses evolved several times through insect evolution, eventually involving strategies for deconstructing plant polymers, detoxifying plant secondary metabolites, and protecting against pathogens (Biedermann and Vega, 2020). Selected throughout insects and microbes’ evolution, these associations may teach many lessons on how to find efficient microbial degraders and detoxifiers of plant tissues, how to assemble an efficient plant-degrading community, and how to promote metabolic interactions for obtaining nutrients from recalcitrant polymers. Investigating the microbial strategies for decaying plant biomass could bioinspire the tuned application of hydrolytic and oxidative pathways to degrade plastics and to generate value-added products (Holladay et al., 2007; Cook and Doran-Peterson, 2010; Huang et al., 2010; Sun and Scharf, 2010; Shi et al., 2011; Koch et al., 2014; Wang et al., 2015; Dangles and Casas, 2019; Tiso et al., 2021).

Relying on microbial associations for utilizing plant-derived resources, fungicultural systems could act as source of microorganisms, metabolic pathways, and microbial interactions eventually participating in the depolymerization of synthetic plastics. Here, we postulate that the microbial plant-degrading systems at the core of insect fungicultures are promising candidates for bioremediation research. First, we compare the degradation of lignocellulose and plastic polymers, highlighting the overlapping microbial players and enzymatic activity between these processes. Second, we review the literature on the metabolic potential of fungiculture associated microbes, focusing on features that, while supporting fungiculture ecology and evolution, could also be applied in biotechnological processes. Third, we suggest multidisciplinary strategies to explore the metabolic potential of fungiculture microbial consortia based on microbial interactions and interdependencies. Fungicultural metabolic landscape could inspire biomimetic approaches, joining the efforts for mitigating the current environmental degradation through a circular economy.

## Microbial Depolymerization of Plastics Trough Pathways for Degrading Plant Polymers

The annual worldwide production of synthetic plastics comprises several hundred million tons of wide range of high molecular weight polymers. In 2015, around 388 million tons of plastics were produced, with 1,722 billion Euro of estimated annual revenue (United Nations Environment Programme Technical University of Denmark [DTU], 2018). Petroleum-derived polyethylene (PE), polypropylene (PP), polystyrene (PS), polyvinylchloride (PVC), polyurethanes (PUR), and polyethylene terephthalate (PET) comprise the majority of plastic polymers currently produced (Figure 1A; Geyer et al., 2017). They are formulated by polyaddition or polycondensation (Eyerer, 2010), and according to their melting properties, are categorized as: (i) Thermoplastics, that can be reshaped by repetitive melting by heating and hardening by cooling, and include PE, PP, PS, PVC, and PET; (ii) Thermosets, that have highly cross-linked chains rendering polymers that cannot be reshaped by heating, as PUR (Zimmermann, 2021). The environmental threat caused by the growing accumulation of plastics makes the search for innovative waste disposal approaches an urgent issue for humankind. Plastic waste disposal is currently done by landfilling (79% of the global disposal), incineration (12%), mechanical and chemical recycling (9%), which present limitations regarding land occupation, toxicity of secondary pollutants, and loss of mechanical properties reducing the plastic’s commercial value, respectively (Garcia and Robertson, 2017; Geyer et al., 2017; Peng et al., 2019; Ru et al., 2020). Plastics entered in the natural landscapes as disposal after the 1960s, and their stability and durability challenge biodegradation processes. Synthetic polymers are thought to take long periods of time to be degraded, particularly due to their high molecular weight, strong C-C bonds, surface hydrophobicity, presenting amorphous and crystalline regions, features that hamper enzymatic attack. Also, conventional plastic products frequently comprise mixtures polymers, solubilizers, plastifiers, pigments, and other chemical compounds that define mechanical properties of plastics and also may further interfere with degradative activities Danso et al., 2019).

Nevertheless, microbial biodegradation of plastic waste has been reported by a number of fungi and bacteria regarded as a promising approach for the removal of environmentally accumulated plastics (Restrepo-Flórez et al., 2014; Danso et al., 2019; Montazer et al., 2020; Ru et al., 2020; Ali et al., 2021a). Plastic biodegradation encompasses processes of bio-deterioration (deriving from microbial biofilms established on the plastic surface and in the interior, altering microstructural and physicochemical properties) and bio-fragmentation (a lytic process relying on the enzymatic activity of surface-colonizing microorganisms, that reduce the polymers molecular weight while releasing oligomers and monomers, Figure 1C; Jacquin et al., 2019; Ali et al., 2021b). As we highlight in the following section, enzymes active during the biodegradation of synthetic plastic polymers include some hydrolases and oxidoreductases related to plant polymers breakdown, such as laccases (EC 1.10.3.2), manganese peroxidases (EC 1.11.1.13), hydroquinone peroxidases (EC 1.11.1.7), alkane hydroxylases (EC 1.14.15.3), cutinases (EC 3.1.1.74), esterases (EC 3.1.1.1), lipases (EC 3.1.1.3), and carboxylesterases (EC 3.1.1.1; Krueger et al., 2015a; Danso et al., 2018).

Several synthetic plastics derive from crude oil monomers, then presenting chemical bonds similar to other natural polymers, as plant polymers. It seems plausible that enzymes that degrade natural plant polymers would also be capable of break down synthetic polymers (Figure 1B; Fich et al., 2016; Chen C.-C. et al., 2020). Plant biomass is composed of non-polyssacharide polymers (as cutin and lignin) and polysaccharide polymers (as cellulose and hemicellulose). Cutin is a hydrophobic polyester composing the outer layer of terrestrial plants that prevents water loss. It is made of epoxide groups and oxygenated fatty acids, which may be branched or linear. Cutinases (a type of serine esterases) are hydrolases that target cutin by catalyzing ester hydrolysis, and have a promising role in breaking synthetic polyesters such as PET. Lignin is a complex heteropolymer composed of aromatic subunits united by C-O and C-C bonds, which also bond the subunits of most of plastic polymers. Therefore, elucidating microbial depolymerization of lignin, as well as the metabolism of aromatic subunits, could unveil mechanisms for degrading synthetic polymers. Lignin-modifying enzymes act by non-specific, oxidative mechanisms, that trigger and accelerate reactive oxygen chain reactions where free radicals decompose lignin and include laccases, lignin peroxidases, manganese peroxidases, dye-decolourizing peroxidases, versatile peroxidases, unspecific peroxidases, and laccases (Yang et al., 2013a; Karich et al., 2017; Chukwuma et al., 2020; Liu et al., 2021; Zhuo and Fan, 2021; Dhagat and Jujjavarapu, 2022). Laccases (EC 1.10.3.2) are multicopper oxidases that use O_2_ as electron acceptor for oxidizing phenolic substrates, though having redox potentials (0.5–1.0 V) not strong enough to oxidize non-phenolic subunits. Alternatively, when operating in a laccase-mediator system, the laccase oxidizes a mediator (i.e., a small aromatic compound), that in turn oxidize the non-phenolic substrate (Solomon et al., 1996; Hilgers et al., 2018). Haem-holding peroxidases, as lignin peroxidase (LiP, EC 1.11.1.14), manganese peroxidases (MnP EC 1.11.1.13), and versatile peroxidase (VP, EC 1.11.1.16), catalyze oxidations by employing H_2_O_2_ as co-substrate. While MnPs have the redox potential (1.0–1.2 V) enough to oxidize only phenolic subunits, LiPs and VPs oxidizing redox cofactors (1.4–1.5 V) may act on both phenolic and non-phenolic substrates (Hofrichter, 2002; Pérez-Boada et al., 2005; Martínez, 2007; Ruiz-Dueñas and Martínez, 2009; Mate and Alcalde, 2017). Lignin-oxidizing enzymes also include dye-decolourizing peroxidase (DyP, EC 1.11.1.19) and chloroperoxidase (CPO, EC 1.11.1.10), comprising haem-holding peroxidases without phylogenetic relationship with other ligninolytic peroxidases. DyP and CPO exhibit a redox potential (1.2–1.5 V) high enough to oxidize phenolic and non-phenolic lignin, and have been employed in several detoxification processes (Husain and Qayyum, 2013; Wang et al., 2018; Chen C.-C. et al., 2020). Lignin is also degraded by Fenton chemistry based on hydroquinone redox processes, pathways that are important for wood decay by brown-rot fungi. For degrading lignocellulose through such mechanism, aryl alcohol oxidases act on aromatic alcohols producing hydrogen peroxide (H_2_O_2_) for the Fenton reactions. These take place when hydrogen peroxide reacts with substrate-derived reduced iron (Fe^2+^), resulting in hydroxyl radicals that break the chemical bonds that provide the recalcitrant nature of lignin. Microbial produced hydroquinones are supposed to reduce the substrate-derived Fe^3+^ to Fe^2+^, then feeding the cycle (Goodell et al., 1997; ten Have and Teunissen, 2001; Suzuki et al., 2006; Arantes et al., 2011, 2012; Eastwood et al., 2011; Schiøtt and Boomsma, 2021).

Cellulose is a polysaccharide-based polymer with high molecular weight, composed of D-glucopyranose units linked by β-1,4-glycosidic bonds, structured as bunches of microfibrils. These are linked through intra- and intermolecular H-bonds and hydrophobic interactions, ultimately forming amorphous and crystalline compacted regions (Park et al., 2010). As for cellulose, synthetic polymers also feature dense and stable crystalline regions, which further impose limitations for enzymatic degradation (Wei and Zimmermann, 2017). Thus, microbial strategies to overcome the structural challenges imposed by crystalline regions could also be applied to synthetic polymers (Chen C.-C. et al., 2020; Daly et al., 2021). For instance, lytic polysaccharide monooxygenases (LPMOs, EC 1.14.99.53–56) reduce Cu^2+^ to Cu^+^ using exogenous electrons, then reacting with O_2_ to form a copper–superoxide complex that deconstruct crystalline cellulose. Such activity split apart the microfibrils, releasing oxidized carbohydrates, and providing access to cellulases (as glycoside hydrolases, GHs) that catalyze the hydrolysis of glycosidic bonds (Vaaje-Kolstad et al., 2010; Bertini et al., 2018; Frommhagen et al., 2018a,b; Song et al., 2018; Liu et al., 2021). For not having substrate specificity, LPMOs may bind and depolymerize other polysaccharidic polymers, such as chitin, xylan, and hemicellulose (Vaaje-Kolstad et al., 2010; Agger et al., 2014; Simmons et al., 2017). In addition, enzymes catalyzing depolymerization of recalcitrant molecules tend to share some features: (i) An extensive and/or flexible active site which allows long-chain polymers to bind; (ii) A flat active site that could facilitate substrate-binding: (iii) Low molecular weight, making these extracellular proteins reduced enough to cross dense polymeric matrices. Membrane proteins may also aid in hydrophobic interactions between the microbial cell and the hydrophobic surface of the polymer (Chen C.-C. et al., 2020). Since hydrolytic and oxidative activities are required for degrading both plant and synthetic plastic polymers, these enzymatic systems are considered applicable for plastic waste recycling and valorization, once more components and mechanisms are discovered and engineered (Chen C.-C. et al., 2020; Zhu et al., 2022).

Water-proof function renders cutin, lignin, and plastic polymers highly hydrophobic physical-chemical properties that also interfere with microbial colonization and degradation. Hydrophobicity, together with other surface physicochemical properties such as roughness, charge, area, and topography, determines which microorganisms would be able to colonize and degrade the polymer (Fich et al., 2016; Cai et al., 2019; Daly et al., 2021). Mechanisms that facilitate microbial attachment to hydrophobic surfaces may mediate hydrophobic interactions allowing the adhesion, ultimately aiding to the degradation processes. Adhesion mechanisms may rely on the tendency of non-polar components to aggregate in water solution, forming “hydrophobic bonds” that reduce the hydrocarbon-water interface area, thus allowing microorganism-surface adhesive interactions (Breslow, 1991; Doyle, 2000; Tribedi and Sil, 2013; Zettler et al., 2013; Mangwani et al., 2015). Bacterial hydrophobic components include emulsan, peptidoglycan, mycolic acids, fimbrial proteins, lipopolysaccharide, lipoteichoic acid, phospholipids, CSh-A and other surface proteins (Doyle, 2000). Fungal hydrophobins are surface hydrophobic proteins that set up fungal aerial structures and intermediate the hyphal adherence to hydrophobic surfaces, being recognized as potential bioremediation tools (Wösten and Wessels, 1997; Sánchez, 2020). On hydrophobic-hydrophilic interfaces, fungal hydrophobins self-assemble as amphipathic monolayers allowing for strong adhesion, increased surface and hydrolysis activity. For instance, the hydrophobin RolA extracted from *Aspergillus oryzae* enhanced PET hydrolysis, possibly by making PET surface more hydrophilic, therefore more susceptible to hydrolytic attack (Sánchez, 2020; Puspitasari et al., 2021). High cell surface hydrophobicity enhanced the attachment of *Pseudomonas* sp. AKS2 to the hydrophobic surface of LDPE, suggesting that biofilm formation may be related to hydrophobic interactions and higher degradation of synthetic polymers (Tribedi et al., 2012; Tribedi and Sil, 2013).

Promoting the community adhesion to the plastic surface, microbial biofilms are essential for processes of plastic colonization, deterioration, and degradation. Biofilms comprise microbial communities enclosed in a self-secreted matrix composed of extracellular polymeric substances, from which unique properties emerge (Flemming et al., 2016). These properties include sorption of enzymes and toxins, niche compartmentalization, and syntrophic interactions allowing for biodegradation networks to be built (Edwards and Kjellerup, 2013; Flemming et al., 2016; Leng, 2017; Sivadon et al., 2019). Acting as a sponge, biofilms could retain and accumulate enzymes in close proximity to the hydrolysis site, rendering the entire structure with degradative activity. Hydrolysis products could concentrate throughout a gradient promoting niche compartmentalization, likely assembling together microbial partners with complimentary metabolism (Pelz et al., 1999; Pazos et al., 2003; Mann and Wozniak, 2012; Harrington and Sanchez, 2014; Flemming et al., 2016; Cavaliere et al., 2017). Biofilm formation, specially investigated in aquatic environments, is influenced by plastic’s physical-chemical features (as hydrophobicity) and roughness. With plastic being a substrate for microbial colonization, biofilm is involved in ecological succession and trophic interactions, thought to mediate plastic degradation (Yuan et al., 2020).

While plant components have been used by microorganisms as resource over millions of years (Floudas et al., 2012), plastic polymers are present in natural ecosystems over some decades, not enough for driving the evolution of mechanisms targeting specifically all these compounds. Also, many of petroleum-derived plastics lack hydrolyzable functional groups and oxidized components. Plastics depolymerization consequently requires higher redox potential than the observed for most of oxidoreductases, thus more recalcitrant to degradation (Krueger et al., 2015a). Notwithstanding, the efficient enzymatic system that evolved to utilize plant polymers as resource seem to be employed by microorganisms to break down synthetic plastics (Chen C.-C. et al., 2020; Mohanan et al., 2020; Sánchez, 2020; Daly et al., 2021; Cowan et al., 2022). The apparent adaptation of preexisting hydrolytic and oxidative pathways suggest that plant and plastic polymers share, in some extent, structural and physical-chemical properties, which is useful for biorremediation (Mueller, 2006; Krueger et al., 2015a; Ali et al., 2021a; Daly et al., 2021). Some of the overlapping mechanisms for deconstruction of plant and synthetic plastics are summarized in the following sections, where we focus on microbial players and enzymatic pathways related to degradation of C-C backbone plastics (PE, PP, PS, PVC) and heteroatomic backbone plastics (PUR and PET).

### Microbial Degradation of C-C Backbone of Plastics

Polyethylene, polypropylene, polystyrene, and polyvinyl chloride are the most abundantly produced synthetic polymers (Figure 1A). Composed exclusively of carbon atoms and not attached to reactive groups, these polymers lack hydrolyzable bonds that would allow hydrolytic degradation. For being non-hydrolyzable, their initial depolymerization relies on redox reactions that release oligomers of lower molecular weight. These may be utilized by microorganisms, entering in diverse metabolic pathways (Krueger et al., 2015a).

#### Polyethylene Depolymerization

Polyethylene (PE) is composed by long chains of ethylene polymerized into various forms, in special low-density PE (LDPE) and high-density PE (HDPE), that differ regarding branching, molecular packing, crystallinity, and density (Danso et al., 2019; Ru et al., 2020; Cowan et al., 2022). PE long C-H chains present high stability and balanced charges that together with the high molecular weight, impose limitations to microbial degradation. This requires local electric charge destabilization, which tend to be achieved by oxygenases that incorporate oxygen to long carbon chains (Krueger et al., 2015a). When PE is oxidized, carboxylic groups, ketones, alcohols and aldehydes are formed, increasing the polymer hydrophilicity and facilitating lipases and esterases to access carboxylic groups, and endopeptidases to access amide groups (Vasile, 2005; Gewert et al., 2015). PE degrader strains have been isolated from marine water, oil-contaminated soil, sewage sludge, and landfills (Ru et al., 2020). Bacterial strains reported to modify and degrade PE include *Pseudomonas aeruginosa, P. putida, P. syringae* (Kyaw et al., 2012; Pramila et al., 2012; Yoon et al., 2012; Tribedi and Sil, 2013), *Rhodococcus ruber* (Orr et al., 2004; Gilan and Sivan, 2013), *Bacillus* sp., *Bacillus subtilis, Bacillus cereus, Bacillus sphaericus, Bacillus pumilus, Bacillus amyloliquefaciens* (Sudhakar et al., 2008; Harshvardhan and Jha, 2013; Yang et al., 2014; Das and Kumar, 2015); *Enterobacter asburiae* (Yang et al., 2014); *Serratia marcescens* (Azeko et al., 2015); *Achromobacter xylosoxidans, Zalerion maritimum* (Kowalczyk et al., 2016); *Brevibacillus parabrevis, Acinetobacter baumannii* (Pramila et al., 2012), *Comamonas* sp., *Delftia* sp., *Stenotrophomonas* sp. (Peixoto et al., 2017). Fungal PE degraders comprise *Aspergillus* sp. *Aspergillus versicolor, Aspergillus flavus, Aspergillus niger* (Manzur et al., 2004; Pramila and Ramesh, 2011a,b; Sowmya et al., 2012; Sheik et al., 2015); *Chaetomium* sp. (Sowmya et al., 2012); *Penicillium simplicissimum, Penicillium pinophilum*, *Penicillium chrysosporium* (Yamada-Onodera et al., 2001; Manzur et al., 2004; Sowmya et al., 2015a,b), *Lasiodiplodia theobromae, Paecilomyces lilacinus* (Sheik et al., 2015), *Trichoderma harzianum* (Sowmya et al., 2014), and *Gliocladium virens* (Manzur et al., 2004). PE degradation were also recognized in the gut of the waxworms T. virens (= *Gliocadium virens*) (Yang et al., 2014; Bombelli et al., 2017), *Achroia grisella* (Kundungal et al., 2019), and *Plodia interpunctella* (Yang et al., 2014, 2015a). Waxworm’s gut microbiota is hypothesized to take part in the degradation process, as exemplified by the PE-degrading capacity of *Enterobacter asburiae* YT1 and *Bacillus* sp. YP1 isolated from *P. interpunctella* gut (Yang et al., 2014, 2015a).

Despite the abundance of PE-degrader microbes, metabolic pathways for PE degradation are not completely elucidated (Ru et al., 2020; Othman et al., 2021). LDPE degradation is hypothesized to involve two stages: (i) Extracellular depolymerization, where LDPE is cleaved into oligomers, dimers, and monomers. Laccase and alkane hydrolase activities seem to be significant during this step; (ii) PE shorter chains may cross the microbial plasmatic membrane to be mineralized into end products as CO_2_, H_2_O, and CH_4_, used as carbon sources for diverse metabolic pathways (Sen and Raut, 2015). The oxidative activity of laccase facilitates cleaving amorphous regions of HDPE (Kang et al., 2019; Ghatge et al., 2020). While the extracellular laccase secreted by *Rhodococcus ruber* C208 oxidized PE, generating carbonyl groups and decreasing molecular weight (Santo et al., 2013), manganese peroxidase (MnP) from the ligninolytic fungi *Phanerochaete chrysosporium* caused a decrease in PE molecular weight and tensile strength (Iiyoshi et al., 1998). Also, LDPE degradation was reported for recombinant *Escherichia coli* expressing alkane hydroxylase genes (*alkB, alkB1*, and *alkB2*), indicating the importance of these genes in PE degradation pathways (Yoon et al., 2012; Jeon and Kim, 2015, 2016). Oxidized carboxylic molecules are converted into acetyl -CoA or propionyl -CoA by β-oxidation, the latter being carboxylated into succinyl -CoA by a propionyl-CoA carboxylase. Both acetyl -CoA and succinyl coA are channeled into the tricarboxylic acid cycle (TCA cycle; Gravouil et al., 2017; Jacquin et al., 2019). Indeed, *Rhodococcus rhodochrous* incorporated oxidized PE oligomers by carriers of the Major Facilitor Superfamily (MFS) or ATP binding cassettes (Eyheraguibel et al., 2017).

#### Polypropylene Depolymerization

Polypropylene (PP) is produced by the polymerization of propylene, forming a straight carbon chain with a hydrophobic surface. Presenting hydrophobic properties, rough surface, and high thermal stability, PP is more resilient to biodegradation than PE (Danso et al., 2019; Othman et al., 2021; Zimmermann, 2021). Potential bacterial PP degraders include *Pseudomonas stutzeri, B. subtilis, Bacillus flexus* (Arkatkar et al., 2010), *Stenotrophomonas panacihumi* (Jeon and Kim, 2016), *Aneurinibacillus aneurinilyticus, Brevibacillus agri, Brevibacillus* sp., *Brevibacillus brevis* (Skariyachan et al., 2018), *Bacillus* sp. strain 27, and *Rhodococcus* sp. strain 36 (Auta et al., 2018). The fungi *P. chrysosporium* and *Engyodontium album* reduced the molecular weight of pretreated PP (Jeyakumar et al., 2013), and *A. niger* may colonize pretreated PP (Alariqi et al., 2006). Even though PP weight loss was reported as indicative of biodegradation in most cases, it is not clear whether it derived from the plasticizer or the C-backbone degradation (Ru et al., 2020). No enzymes, metabolic pathways, and microbial mechanisms for PP biodegradation were described so far (Arutchelvi et al., 2008; Danso et al., 2019; Chandra and Singh, 2020; Kumar et al., 2020).

#### Polystyrene Depolymerization

Polystyrene (PS) is an aromatic synthetic compound resulting from the polymerization of an aromatic styrene monomer. This aromatic polymer persists in the environment due to its high molecular weight and hydrophobicity, besides being hard and rigid (Othman et al., 2021; Zimmermann, 2021). Bacterial strains reported to participate in PS degradation include *Xanthomonas* sp., *Sphingobacterium* sp., *Bacillus* sp. STR-YO (Oikawa et al., 2003), *P. putida* CA-3 (Ward et al., 2005), *P. aeruginosa* (Atiq et al., 2010), *Rhodococcus ruber* C208 (Mor and Sivan, 2008), *Microbacterium* sp. NA23, *Paenibacillus urinalis* NA26, *Bacillus* sp. NB6, *B. subtilis, Staphylococcus aureus, Streptococcus pyogenes* (Asmita et al., 2015). Some degradation of PS was achieved by microbial consortia on soil and liquid enrichment cultures, possibly relying on oxidative reactions carried out by bacterial genera such as *Bacillus, Pseudomonas, Micrococcus*, and *Nocardia* (Sielicki et al., 1978). Fungal degradation was observed for the strains *Curvularia* sp. (Motta et al., 2009), *Rhizopus oryzae* NA1, *Aspergillus terreus* NA2, *P. chrysosporium* NA3 (Atiq, 2011). Limited degradation was accomplished by a fungal consortia consisting of strains of *Coriolus hirsutus, Gloeophyllum trabeum, Coriolus versicolor, Bjerkandera adusta, Daedalea quercina, Phellinus pini, Aureobasidium pullulans, Fomes annosus, Peniophora gigantea, Fomes everhartii, Poria xantha, A. fumigatus, Paecilomyces varioti, Trichoderma koningii*, and *A. niger* (Kaplan et al., 1979). The white rot fungi *Pleurotus ostreatus, P. chrysosporium*, and *Trametes versicolor* were able to degraded PS-lignin copolymers (Milstein et al., 1992). However, laccase isolated from *T. versicolor* depolymerized the synthetic polymer polystyrene sulfonate (PSS) only when the mediators ρ-coumaric acid, syringaldehyde, and the synthetic mediator 1-HBT were added. On the other hand, the brown-rot basidiomycete *Gloeophyllum trabeum* depolymerized PSS *via* extracellular hydroquinone Fenton chemistry, through a seemingly unspecific process where the polymer was randomly cleaved throughout the chain (Krueger et al., 2015b,^2017^).

Polystyrene biodegradation is initiated by microbial biofilm that attach and partially degrade the polymer surface, as reported for *R. ruber* (Mor and Sivan, 2008) and *Exiguobacterium* sp. DR11 and DR14 (Chauhan et al., 2018). Biodegradation pathways vary depending on the participating microorganism, since diverse bacterial strains metabolize the monomer styrene, including *Pseudomonas, Xanthobacter, Rhodococcus*, and *Corynebacterium* (Ho et al., 2018; Danso et al., 2019). Polystyrene backbone is hypothesized to be degraded by hydrolases, resulting in styrene monomers (Othman et al., 2021). So far, only hydroquinone peroxidase produced by the lignin-degrader *Azotobacter beijerinckii* HM121 was reported to depolymerize PS into metabolites of low molecular weight (Nakamiya et al., 1997). On the other, the monomer styrene is oxidized by two pathways: (i) Attack of an unspecific aromatic ring, catalyzed by a dioxygenase and by a dihydrodiol dehydrogenase, resulting in the intermediates 3-vinylcatechol, phenylacetic acid, and 2-phenylethanol, which are directed into the Krebs cycle. (ii) Oxidation of the vinyl side chain by a styrene monooxygenase that releases epoxystyrene, which is isomerized by a styrene oxide isomerase to form phenylacetaldehyde, which is then oxidized into phenylacetic acid by a phenylacetaldehyde dehydrogenase. Phenylacetic acid is converted to phenylacetyl coenzyme A, that forms acetyl-CoA after β-oxidation, which then enters in the TCA cycle (Tischler et al., 2009; Tischler, 2015; Danso et al., 2019; Jacquin et al., 2019). *P. putida* and *Rhodococcus zopfii* convert polystyrene (thermally transformed into styrene oil) into polyhydroxyalkanoate, a value-added biodegradable polymer (O’Leary et al., 2005; Ward et al., 2005, Ward et al., 2006). Curiously, the larvae of *Tenebrio molitor* and other mealworms, dark mealworms (*Tenebrio obscurus*), and superworms (*Zophobas atratus*) eat and degrade PS, which seems to be assisted by the gut microbiota in some extent (Yang et al., 2015b,c, 2018, 2020; Brandon et al., 2018). For instance, PS weight loss was achieved by *Exiguobacterium* sp. YT2 isolated from *T. molitor* gut (Yang et al., 2015c).

#### Polyvinyl Chloride Depolymerization

Polyvinyl chloride (PVC) is a high molecular weight synthetic polymer composed of vinyl chloride monomers, highly hydrophobic and resilient (Shah et al., 2008b; Ali et al., 2021a). PVC presents high proportions of plasticizers (up to 50%), that may be a nutritional source for bacteria and fungi. Even that plasticized PVC is susceptible to microbial degradation, the decrease in PVC weight loss probably resulted from plasticizer degradation rather than the PVC chains (Ali et al., 2021a; Zimmermann, 2021). Both microbial degraders and metabolic pathways able to fully depolymerize PVC-plasticizer have not been reported (Ru et al., 2020). Some microorganisms that seem related to PVC biodegradation include the bacterial strains *Mycobacterium* sp. NK0301 (Nakamiya et al., 2005); *Chryseomicrobium imtechense, Lysinibacillus fusiformis, Acinetobacter calcoaceticus, Stenotrophomonas pavanii* (Latorre et al., 2012), *Acanthopleurobacter pedis, Bacillus cereus, Bacillus aerius* (Shi et al., 2011; Anwar et al., 2016), *Bacillus flexus* (Giacomucci et al., 2019), *Bacillus* sp. AIIW2 (Kumari et al., 2019), *Pseudomonas otitidis* (Shi et al., 2011; Anwar et al., 2016), *P. aeruginosa, P. putida, Pseudomonas citronellolis* (Shi et al., 2011; Giacomucci et al., 2019), *Microbacterium* sp. and *Bacterium* Te68R (Shi et al., 2011). PVC degradation was accomplished in some extent by the fungal strains *Alternaria* sp. TOF-46 (Moriyama et al., 1993), *Trametes versicolor*, *Pleurotus sajor-caju* (Kırbaş et al., 1999), *Aureobasidium pullulans* (Webb et al., 1999, 2000), *A. niger* (Gumargalieva et al., 1999; Ali et al., 2014; Giacomucci et al., 2019); *Penicillium janthinellum* (Sabev et al., 2006), *Phanerochaete chrysosporium* (Ali et al., 2014; Khatoon et al., 2019), *Lentinus tigrinus*, and *A. sydowii* (Ali et al., 2014). The gut microbiota of *T. molitor* larvae was supposed to participate in PVC depolymerization, which was partially mineralized to chloride (Peng et al., 2020).

### Heteroatomic Polymers

Having a heteroatomic backbone, polyethylene terephthalate and polyurethane are linked by ester and urethane bonds, respectively. These polymers are susceptible to hydrolysis, resulting in oligomers and carboxylic end groups (Krueger et al., 2015a; Mohanan et al., 2020).

#### Polyethylene Terephthalate

Polyethylene terephthalate (PET) is a polar and linear thermoplastic, constituted by repeated molecules of aromatic terephthalic acid and ethylene glycol united by ester bonds. The resulting bis (2-hydroxyethyl) terephthalate (BHET) is the PET monomeric unit (Webb et al., 2013; Danso et al., 2019; Zimmermann, 2021). PET is a semicrystalline polymer, comprising crystalline regions that are resistant to enzymatic attack. Degradation of polymeric chains requires enough flexibility for allowing enzymatic attack. Therefore, amorphous regions are supposedly attacked first, rendering crystalline regions prone to enzymatic activity. PET amorphous regions are, however, susceptible to hydrolysis, and microbial enzymes identified for PET degradation include PET hydrolase and tannase, and serine hydrolases as cutinases and lipases (Wei and Zimmermann, 2017; Danso et al., 2018; Kawai et al., 2019; Zimmermann, 2021). PET depolymerization has been reported for the bacterial strains *Bacillus amyloliquefaciens* (Novotný et al., 2018), *Ideonella sakaiensis* (Yoshida et al., 2016; Wei et al., 2019a), *Nocardia* sp. (Sharon and Sharon, 2012), *Pseudomonas mendocina* (Ronkvist et al., 2009), *Saccharomonospora viridis* (Kawai et al., 2014), *Thermobifida fusca* (Müller et al., 2005; Wei et al., 2019b), *Thermomonospora fusca* (Alisch et al., 2004), *Yarrowia lipolytica* (da Costa et al., 2020). Fungal strains also exhibited PET depolymerizing capacity, such as *Aspergillus* sp. (Sarkhel et al., 2020), *Fusarium oxysporum* (Nimchua et al., 2007), *Fusarium solani* (Alisch et al., 2004; Nimchua et al., 2007), *Penicillium citrinum* (Liebminger et al., 2007), *Penicillium funiculosum* (Nowak et al., 2011), *Penicillium* sp. (Sepperumal et al., 2013), engineered *Pichia pastoris* (Chen Z. et al., 2020), *Thermomyces insolens* (formerly *Humicola insolens;* Ronkvist et al., 2009), *Thermomyces lanuginosus* (Fernandez-Lafuente, 2010), and *Thielavia terrestris* (Yang et al., 2013b).

Polyethylene terephthalate depolymerization involves both the modification of surface polyester fibers and hydrolysis of the inner bulk, and these processes are carried out by different enzymes with distinct properties. PET surface-modifying enzymes include lipases, carboxylesterases, cutinases, and proteases (Kawai et al., 2019). These hydrolases may modify surface components producing polar hydroxyl and carboxylic groups, though without degrading PET inner bulk, as exemplified by the cutinase-like enzymes PmC from *P. mendocina* and FsC from *F. solani* (Ronkvist et al., 2009; Kawai et al., 2019). The hydrolysis of PET building blocks is an outcome from the flexibility of the polymer chain and structural properties of the enzyme (particularly the accessibility of the active site to the polymer surface; Zumstein et al., 2017; Kawai et al., 2019). PET hydrolases could lead to substantial degradation of PET building blocks (Kawai et al., 2019), as reported for the cutinase-like hydrolases TfH from *Thermobifida fusca* (Mueller et al., 2005), HiC from *Thermomyces insolens* (Ronkvist et al., 2009), and Cut190 from *Saccharomonospora viridis* AHK190 (Kawai et al., 2014). Esterase activity hydrolyzes PET, releasing, in majority, terephthalic acid (TPA) and ethylene glycol (EG), besides bis-(2-hydroxyethyl) terephthalate (BHET) and mono-(2-hydroxyethyl) terephthalate (MHET), that are subproducts of incomplete hydrolysis. A TPA transporter may lead TPA into the bacterial cell, where the sequential activity of a dioxygenase and dicarboxylate dehydrogenase convert it to protocatechuate. By distinct dioxygenases, protocatechuate may be degraded *via ortho*-, *meta*-, and *para*-cleavage pathways, rendering metabolites that will eventually be converted into acetyl-CoA and succinyl-CoA, which channel into the tricarboxylic acid (TCA) cycle for forming succinic acid (Hosaka et al., 2013; Salvador et al., 2019; Ru et al., 2020). *P. putida* GO16, *P. putida* GO19, and *Pseudomonas frederiksbergensis* GO23 are able to both metabolize and accumulate TPA, polymerizing medium chains of polyhydroxyalkanoate (PHA; Kenny et al., 2008). EG may be metabolized by acetogens pathway, where it is degraded to ethanol and acetaldehyde, then transformed to acetate *via* acetyl-CoA (Trifunović et al., 2016). Alternatively, by the pathway of *Pseudomonas aeruginosa*, a series of dehydrogenases oxidize EG into glycolate, that is oxidized into glyoxylate, converted into glycerate and then into pyruvate, ultimately entering in the TCA cycle (Kataoka et al., 2001; Ru et al., 2020).

A PETase enzyme was identified in *Ideonella sakaiensis* 201-F6 (*Is*PETase), a bacterial strain able to colonize and degrade amorphous PET film in some extent (Yoshida et al., 2016). Related to actinomycete cutinases, PETases (EC 3.1.1.101) hydrolytic activity may vary according to PET crystallinity, thus remaining to be elucidated whether PETase indeed act as PET hydrolase (Kawai et al., 2019). While *Is*PETase may hydrolyze amorphous regions, it is not active against crystalline PET (Yoshida et al., 2016; Kawai et al., 2019; Wei et al., 2019b). Overall, PETase hydrolyze PET into MHET, producing TPA and BHET as secondary products. An enzyme known as MHETase, converts MHET to TPA and EG, both following the metabolic pathways previously described (Yoshida et al., 2016, 2021; Chen et al., 2018; Salvador et al., 2019). Besides, potential PET hydrolases were identified in globally distributed microbial genomes and metagenomes, with the majority of enzyme candidates occurring in the bacterial phyla Actinobacteria, Proteobacteria (Betaproteobacteria, Deltaproteobacteria, and Gammaproteobacteria), and Bacteroidetes (Danso et al., 2018). Potential enzymes acting on polyesters were also identified by genome and metagenomic mining, including a cutinase from *P. pseudoalcaligenes* (PpCutA) and a putative lipase from *Pseudomonas pelagia* (PpelaLip; Haernvall et al., 2017). A hydrocarbon-acclimated microbial consortia initiated PET degradation, where *Alcanivorax* seem an important PET colonizer (Denaro et al., 2020).

#### Polyurethanes

Polyurethanes (PUR) generally designate heteropolymers synthesized from polyol and polyisocyanate subunits united by urethane bonds, though the polymer may also contain ether or ester bonds. Thus, PUR structure is undefined, and urethane bonds may comprise a small proportion of the molecule. According to the polyol chemical structure, PUR may be termed either polyester PUR (when derived from a polyester polyol) or polyether PUR (when derived from a polyether polyol). Therefore, PUR present diverse formulations, conformations and macromolecular architecture, having both crystalline regions that are more recalcitrant to microbial degradation, and amorphous regions more susceptible to enzymatic attack (Howard, 2012; Cregut et al., 2013; Krueger et al., 2015a; Ru et al., 2020; Zimmermann, 2021). Also, due to the chemical bonds, polyester PUR is more susceptible to microbial degradation than polyether PUR (Darby and Kaplan, 1968). An increasing number of microbial strains have been reported as PUR degraders (Cregut et al., 2013; Danso et al., 2019), including the bacterial strains: *Acinetobacter gerneri* (Howard, 2012), *Alicycliphilus* sp. BQ1 (Oceguera-Cervantes et al., 2007), *Arthrobacter* sp. AF11 (Shah et al., 2008a), *Bacillus* sp. (Ii et al., 1998), *Bacillus* sp. AF8 (Shah et al., 2016), *B. subtilis* (Rowe and Howard, 2002; Shah et al., 2008a,^2013^, Koraichi, 2015; Stepien et al., 2017), *Bacillus safensis* (Nakkabi et al., 2015), *Bacillus pumilus* (Nair and Kumar, 2007), *Comamonas acidovorans* (Nakajima-Kambe et al., 1995), *Corynebacterium* sp. BI2 (Kay et al., 1991), *Micrococcus* sp. 10 (Shah et al., 2008a), *Pseudomonas* sp. AF9, *P. aeruginosa* (Shah et al., 2016), *Pseudomonas denitrificans, P. fluorescens* (Howard and Blake, 1998; Stepien et al., 2017), *P. putida* (Peng et al., 2014), *Pseudomonas chlororaphis* (Howard et al., 1999), *Pseudomonas chlororaphis* (Gautam et al., 2007), and *Staphylococcus epidermidis* (Jansen et al., 1991). Fungal PUR degraders encompass *Alternaria* sp. PURDK2 (Matsumiya et al., 2010), *Alternaria* sp. (Magnin et al., 2019), *Alternaria tenuissima* (Oprea et al., 2018), *Aspergillus* sp. S45 (Osman et al., 2018), *Aspergillus* sp. (Magnin et al., 2019), *A. flavus* (Mathur and Prasad, 2012), *A. niger* (Filip, 1979), *Aspergillus tubingensis* (Khan et al., 2017), *Chaetomium globosum* (Darby and Kaplan, 1968), *Cladosporium herbarum* (Filip, 1979), *Cladosporium tenuissimum* (Álvarez-Barragán et al., 2016), *Curvularia senegalensis* (Crabbe et al., 1994), *Geomyces pannorum* (Cosgrove et al., 2007), *Penicillium* sp. (Magnin et al., 2019), *Pestalotiopsis microspora* (Russell et al., 2011), *Phoma* sp. (Cosgrove et al., 2007), and *Yarrowia lipolytica* (Stepien et al., 2017). Concomitant to changes in the gut enzymatic activity and microbiome composition, PUR degradation was observed in the gut of the *Z. atratus*, correlated to the dominance of the bacterial genera *Enterococcus* and *Mangrovibacter* (Luo et al., 2021).

Microbial degradation is driven by PUR properties determining the accessibility of degrading systems, which involves polymer crystallinity, molecular orientation, crosslinking, and chemical groups (Howard, 2002, 2012). PUR-degrading activity was reported for a polyester cutinase (Crabbe et al., 1994), polyester esterases (Akutsu et al., 1998; Allen et al., 1999; Vega et al., 1999; Howard et al., 2001, 2007, 2012; Russell et al., 2011), a membrane bound esterase (Nakajima-Kambe et al., 1995), PueB and PueA lipases from P. chlororaphis (Stern and Howard, 2000, Howard et al., 2001, 2007), Stern and Howard (2000), Howard et al. (2001, 2007), and polyether urethane hydrolases (Owen et al., 1996; Akutsu-Shigeno et al., 2006). Characterized polyurethanases includes both membrane-bound and secreted enzymes that seems to act complementarily in a way that more metabolites may be accessed by microorganisms (Akutsu et al., 1998). PUR degradation by membrane-bound polyurethanases seems a two-step process, where a membrane-bound enzyme adhere to the PUR surface *via* hydrophobic-PUR-surface binding domain. Once bounded to the substrate, the enzyme catalytic domain hydrolyzes urethane bonds and releases polyurethane subunits. Such substrate binding allows the concentration of enzymes close to the substrate, accelerating biodegradation rates. Membrane-bound enzymes are a mechanism for dealing with the non-soluble nature of polyurethanes, which makes secreted enzymes not efficient at substrate binding. Extracellular soluble esterases would further hydrolyze the metabolic products of membrane-bound enzymes, complementing polymer degradation (Howard, 2002; Cregut et al., 2013). For the polycaprolactone polyol-based PUR, an esterase (E3576) could hydrolase ester bonds forming 6-hydroxyhexanoate, that is further metabolized trough alternative pathways to form acetyl-CoA, then entering the TCA cycle (Magnin et al., 2019; Ru et al., 2020).

## Plant-Degrading Microbial Communities From Insect Fungiculture

Plastic-degrading microbes have been reported in several marine and terrestrial contaminated environments, both by culturing and metagenomic methods (Danso et al., 2019; Jacquin et al., 2019; Ru et al., 2020; Yuan et al., 2020). In unexpected environments such as cow rumen, dung, moss, and even guts of larvae and adult insects, enzymes and microbial players were found to have plastic-biodegrading potential. There, the associated microbiota seems to employ some of the enzymatic mechanisms for deconstructing plant biomass to degrade synthetic plastics, based on some chemical and structural similarities between these polymers. Hence, plant-degrading and host-associated microbial communities have been investigated as source of enzymes and/or microbial consortia to potentially compose strategies for biodegrading plastic waste (Yang et al., 2015b,c, 2018, 2020; Müller et al., 2017; Skariyachan et al., 2017, 2021; Peng et al., 2020; Quartinello et al., 2021). Esterases from cow (*Bos taurus*) rumen were able to partially hydrolyze the polyesters PET, polybutylene adipate-co-terephthalate (PBAT, biodegradable) and polyethylene furanoate (PEF, biobased). Polyester degradation in rumen is thought to rely on a microbial community dominated by *Pseudomonas* spp., reported to present diverse hydrolytic activity (Quartinello et al., 2021). Bacterial consortia enriched from cow dung, containing the degrading strains *Bacillus vallismortis* bt-dsce01, *Pseudomonas protegens* bt-dsce02, *Stenotrophomonas* sp. bt-dsce03, and *Paenibacillus* sp. bt-dsce04, partially degraded LDPE and HDPE under thermophilic conditions (Skariyachan et al., 2017). Also obtained from cow dung, consortia composed of *Enterobacter* sp. btDSCE-01, *Enterobacter cloacae* btDSCE-02, and *Pseudomonas aeruginosa* btDSCE-CD03 partially degraded LDPE and PP (Skariyachan et al., 2021). Esterases from the microbiome associated with *Sphagnum magellanicum* moss hydrolyzed polybutylene adipate-co-butylene terephthalate (PBAT) and substrate bis(4-[benzoyloxy]butyl) terephthalate, highlighting the potential of plant-associated microbiomes as source of polymer degrading enzymes (Müller et al., 2017).

The capacity of insect larvae to penetrate and deteriorate plastic is known for a long date, and their promising plastic-degrading potential have gained attention in the last decade, since PS, LDPE, PP, and PVC may be biodegraded after larvae ingestion. Larvae-mediated depolymerization was achieved in the guts of Tenebrionidae beetles (*T. molitor, T. obscurus, Z. atratus, Tribolium castaneum, Uloma* sp., and *Plesiophthalmus davidis*) and Pyralidae moths (*P. interpunctella*; *Achroia grisella; G. mellonella;* Gerhardt and Lindgren, 1954; Cline, 1978; Yang et al., 2014, 2015a; Bombelli et al., 2017; Brandon et al., 2018; Kundungal et al., 2019, 2021; Peng et al., 2019, 2020; Wang et al., 2020; Woo et al., 2020). In the rotting wood of forests where *T. molitor*, *T. obscurus*, and *Z. atratus* naturally occurs, the larvae feed on lignocellulosic material as dried leaves (Calmont and Soldati, 2008; Peng et al., 2019, 2020). Stored-food pests *T. castaneum* and *P. interpunctella* also consume plant materials, including wheat, sorghum, and maize (Hamlin et al., 1931; Williams, 1964; Sokal and Sonleitner, 1968). *Achroia grisella G. mellonella*, and *Uloma* sp. are pests of honey bees’ colonies, where they consume wax material (Ellis et al., 2013). Plastic biodegrading potential by insect larvae reiterates that the enzymatic toolkit for degrading plant-derived and recalcitrant polymers could be adapted for biodegrading synthetic polymers (Chen C.-C. et al., 2020). Thus, herbivorous insect hosts appear to be a valuable source of microbial players and enzymes for depolymerizing synthetic plastics, which could be adapted from a plant-degrading microbial community. Insect fungiculture, in particular, gathers plant-degrading microorganisms and metabolic pathways remaining to be completely explored aiming at plastic waste biodegradation for recycling and upcycling.

**FIGURE 2:**
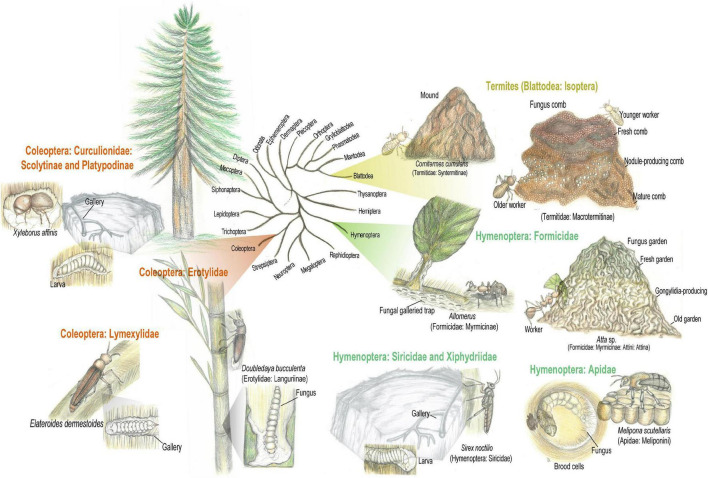
Insect-fungus mutualism evolved in diverse insect orders. Fungal cultivation by insects may occur in two main configurations: (i) Proto-fungiculture, where insects passively propagate fungi that provide either dietary enrichment, protection against pathogens, or structural reinforcement to the colony/nest, presenting few adaptations to maintain the fungal symbiont. Proto-fungiculture have been observed in the lizard beetle *Doubledaya bucculenta* and *Sirex* wood wasps. (ii) Advanced fungiculture, that involve active maintenance of fungal crops by fungus-growing insects, evolving independently in macrotermitine termites, attine ants, platypodinae, and scolytinae beetles. Advanced fungiculture is characterized by high levels of nutritional dependency between fungi and insects fungal crop. Simplified insects phylogeny based on Misof et al. (2014). Pencil drawing illustrations by Mariana Barcoto.

Insect-fungus mutualism evolved in diverse insect orders (Figure 2; Biedermann and Vega, 2020), among which fungus cultivation is considered “a breakthrough innovation in animal evolution” (Wilson, 1986; Mueller and Rabeling, 2008). Fungal cultivation by insects may occur in two main configurations: (i) Proto-fungiculture, where insects passively propagate fungi that provide either dietary enrichment, protection against pathogens, or structural reinforcement to the nest, presenting few adaptations to maintain the fungal symbiont. Proto-fungiculture have been observed in diverse non-social insects, as the lizard beetle *Doubledaya bucculenta* (Toki and Togashi, 2013), and wood wasps in the genera *Sirex* and *Xyphidria* (Kukor and Martin, 1983; Heath and Stireman, 2010; Pažoutová et al., 2010). (ii) Advanced fungiculture, that involve active maintenance of fungal crops by fungus-growing insects, is hypothesized to have arisen during the Paleogene (66–24 Million years ago; Roberts et al., 2016). Such lifestyle evolved independently in termites (Blattodea: Termitidae: Macrotermitinae) between 37–55 Mya, in attine ants (Hymenoptera: Formicidae: Myrmicinae: Attini: Attina) between 55–60 Mya, in ambrosia and bark beetles (Coleoptera: Curculionidae: Scolytinae and Platypodinae) between 90–110 and 1–58 Mya, respectively (Mayhé-Nunes and Jafé, 1998; Mueller et al., 2005; Jordal and Cognato, 2012; Bourguignon et al., 2015; Branstetter et al., 2017; Pistone et al., 2018; Biedermann and Vega, 2020). Advanced fungiculture is characterized by high levels of nutritional dependency between fungi and insects that is maintained by behavioral adaptations for inoculating, cultivating, harvesting, vectoring and cleaning the fungal crop, as well as elaborated waste management (Martin, 1992; Mueller et al., 2005; Biedermann and Vega, 2020). Despite differences in geographic distribution and evolutionary history, advanced insect fungiculture share main ecological features: (i) rearing of the fungal mutualist in architecturally particular structures external to the insect’s body; (ii) the insects provide the fungus with dispersal, protection against (mainly microbial) antagonists, and substrates for nourishment; (iii) mutualistic fungi convert recalcitrant polymeric substrates into more labile energy sources, available to the insects *via* fungal consumption (i.e., mycophagy; Mueller et al., 2005; Biedermann and Vega, 2020). Plant biomass breakdown in fungicultural systems is a gradual and continuous process, following a basic framework that includes substrate pretreatment, lignocellulose degradation, and waste management (Li H. et al., 2021).

Ecological activity of fungus-growing insects may influence wide areas, making them ecosystem engineers that affect geophysical processes, environmental structure, biodiversity, and successional patterns of terrestrial ecosystems (Jones et al., 1997; Dangerfield et al., 1998; Jones, 2012; Meyer et al., 2013; Raffa et al., 2015). By cultivating lignocellulolytic fungal crops, these insect-fungus symbioses are notorious organic matter decomposers, influencing energy and nutrient dynamics over spatial and temporal scales (Abbadie and Lepage, 1989; Jones, 1990; Verchot et al., 2003; Jouquet et al., 2011; Crowther et al., 2012; Siegert et al., 2018; Šamonil et al., 2020). Insects agricultural systems are also inhabited by characteristic, convergent, and adapted microbiota, that appear to integrate pathways for the detoxification of plant defensive metabolites and lignocellulose degradation (Suen et al., 2010; Aylward et al., 2014; Poulsen et al., 2014; Barcoto et al., 2020; Francoeur et al., 2020; Khadempour et al., 2020). The microbiota abundantly encodes genes for xenobiotics modification, such as pathways for polycyclic aromatic carbon and alkane degradation, though the role of these routes are not clear (Barcoto et al., 2020). In the following sections we review the literature on insect fungiculture, especially those based on obtaining nutrient from plant material *via* lignocellulose degradation and detoxifying plant defensive compounds. For cultivating fungi without nutritional implications or in substrates other than plant tissues, other fascinating fungus cultivation systems were not detailed. These include the burying beetles *Nicrophorus vespilloides* (Coleoptera: Silphidae) that maintains a specific microbiota on carrion (Shukla et al., 2018), and the legless mealybug *Orbuspedum machinator* (Hemiptera: Pseudococcidae) that nourishes fungi with honeydew (Gavrilov-Zimin, 2017). Intriguing fungus cultivation systems for which the fungal metabolism of plant components remains to be elucidated were not detailed as well. These encompass the leaf-rolling weevil *Euops chinensis* (Coleoptera: Attelabidae) that cultivates a garden of *Penicillium herquei* for antimicrobial protection of the larvae (Wang et al., 2015), and gall-forming midges (Diptera: Cecidomyiidae) associated with fungi in the family Botryosphaeriaceae (Rohfritsch, 2008; Heath and Stireman, 2010). Whenever possible, we attempted to focus on aspects related to plant-degrading potential of the fungiculture system, to emphasize metabolic pathways that, while supporting insects’ ecology and evolution, could also be applied in biotechnological processes.

### Isoptera-Fungi Mutualisms

#### *Cornitermes cumulans* External Fermentation

Termites (Blattodea: Isoptera) are a dietary diverse group, which have adapted to a variety of food sources including wood, plant litter, herbivore dung, and organic matter highly humified. Since lignocellulose in different stages of decomposition is a consistent diet component for every termite feeding group, digestive strategies employed by termites usually gather enzymatic activity of the host and gut microbiota (Rouland-Lefèvre, 2000; Watanabe and Tokuda, 2010; Jouquet et al., 2011; Ni and Tokuda, 2013; Brune, 2014). Some species of higher termites (Termitidae) evolved associations with external microbial communities that aid in deriving nutrients from plant resources. Certain nesting strategies include building mounds from soil and feces aggregates that may sustain a microbial community and even function as a fermenter system (Korb, 2003, 2011; Fall et al., 2004, 2007; Moreira et al., 2021). Sharing process of plant degradation with nests-associated microbiota is hypothesized to be a fundamental step toward the evolution of Termitidae (Garnier-Sillam et al., 1989; Aanen et al., 2002; Brune, 2014; Moreira et al., 2018; Chouvenc et al., 2021). Although not considered a strictly example of proto-fungiculture, digestive externalization based on microbial degradation of plant material is suggested for *Cornitermes cumulans* (Termitidae: Syntermitinae), where the nest and gut microbiota seem to sustain a complimentary metabolism (Figure 2). This is a grass and litter harvesting termite species that cut and carry plant material into the mound. Once inside the mound, plant material is stored in structures made of saliva and feces, also known as food nodules. Such structures are inhabited in majority by saprotrophs in the fungal orders Pleosporales, Sordariales, and Xylariales (Ascomycota), and bacteria in the phyla Actinobacteria and Proteobacteria. Lignocellulose is initially degraded in food nodules by enzymes assigned to the bacterial phyla Proteobacteria and Actinobacteria, and the fungal phyla Ascomycota. These enzymes target complex polysaccharides as cellulose (fungal endoglucanase EC 3.2.1.4; beta-glucosidases EC 3.2.1.21) and xylan (bacterial xylanase EC 3.2.1.8 and fungal xylanase EC 3.2.1.136), as well as lignin (catalase-peroxidases EC 1.11.1.21; Moreira et al., 2021). Fungal and bacterial lignocellulolytic activity in food nodules possibly pre-treat plant material before the termite gut passage, externally complementing plant digestion (Lima and Costa-Leonardo, 2007; Menezes et al., 2018; Moreira et al., 2021).

#### Advanced Fungiculture of Macrotermitinae Termites

Macrotermitinae termites provide an interesting example of bacterial-fungal complimentary metabolism targeting plant degradation. Macrotermitinae species termites cultivate basidiomycete fungi in the genus *Termitomyces* (*Agaricales*: *Lyophyllaceae*) in a cork-like structure known as fungus comb. As termitomycetoid fungal taxa present a reduced oligosaccharide-degrading enzymatic profile, the fungal metabolism is complimented with gut passages (Rouland-Lefèvre, 2000; Poulsen et al., 2014; Li H. et al., 2017; van de Peppel et al., 2021). Such reduction in plant-degrading enzymes seems to precede domestication by termites and could have even facilitated this process. By targeting lignin and cellulose while stepping oligosaccharides aside, the *Termitomyces* ancestor could supposedly enrich the comb nutritional value, thus favoring the termites (van de Peppel et al., 2021). Therefore, the nutritional role of the fungal crop could be considered as both indirect (by degrading lignin and providing easier access to cellulose and other plant components) and direct (by serving as a food source; Hyodo et al., 2003; Vesala et al., 2019). Nutrition also varies according to the workers’ caste: while the queen and larvae seem to feed on the fungal mycelium, younger workers feed on fungal nodules, adult workers and soldiers obtain energy from plant substrate and the fungus comb (Rouland-Lefèvre, 2000; Nobre and Aanen, 2012; Vesala et al., 2019). *Termitomyces* species are mushroom-forming fungi that also produce conidia (asexual spores) in the mycelium, used to inoculate the substrate and form the comb (Leuthold et al., 1989; Botha and Eicker, 1991; Vreeburg et al., 2020). Hypothesized as an external rumen, the comb is characteristically structured (Figure 2): (i) at the top, the fresh comb has dark color due to freshly added substrate; (ii) at the middle, the mature nodule-producing comb is lighter because of high hyphal density; and (iii) at the bottom, the old comb has high hyphal content, plant biomass almost completely decomposed, and high concentration of oligosaccharides (Rouland-Lefèvre, 2000; Li et al., 2012; Li H. et al., 2017; Nobre and Aanen, 2012; da Costa et al., 2018).

Nutrient dynamics derive from the gut microbiota and workers’ polyethism, as the different tasks performed by older and younger workers set up the substrate processing (Hinze and Leuthold, 1999; Hinze et al., 2002; Li et al., 2015, Li H. et al., 2016). Specific metabolic pathways for lignocellulose metabolism may differ between macrotermitine species, particularly regarding the fate of lignin during the process (Hyodo et al., 2003; Li H. et al., 2017; da Costa et al., 2018, 2019). In general, older workers forage for decaying wood, grass, leaf litter, and herbivorous feces to nourish their fungal crop. Substrates brought into the mound are initially chew up by younger workers, decreasing cellulose crystallinity (Dangerfield and Schuurman, 2000; Aanen et al., 2002; Li et al., 2012, 2015; Li H. et al., 2017). After being ingested, the substrate takes about 3.5 h to transit through the younger workers’ gut. There, in some termite species, lignin sidechains are cleaved and methoxyl rings are removed, possibly assessed by the gut bacterial community (Li H. et al., 2017). Besides lignin depolymerization and metabolism of hemicellulose-derived branched sugars, this first gut passage mixes the plant material with conidia and carbohydrate-degrading enzymes produced by *Termitomyces* in fungal nodules (Rouland-Lefèvre, 2000; Watanabe and Tokuda, 2010; Li et al., 2012; Li H. et al., 2016, 2017; Nobre and Aanen, 2012; da Costa et al., 2018). Such pretreated and conidia-inoculated substrates, free from some lignin subunits, are excreted as the fresh comb (Leuthold et al., 1989; Rouland-Lefèvre, 2000; Li H. et al., 2017). Also, the younger workers’ gut is supposed to originate the fresh comb bacterial community, which could deconstruct and ferment poly- and oligosaccharides (Otani et al., 2016; Li H. et al., 2017). Developing from the fresh to the nodule-producing mature comb takes about 15 to 20 days, increasing nitrogen and carbon content in fungal nodules (Li H. et al., 2017; Hu et al., 2019; Vesala et al., 2019). More than 30 to 35 days are required to achieve the old comb stage (Li H. et al., 2017).

During this turnover time of 45 to 50 days, degradation of additional lignin, cellulose, and hemicelluloses are carried out by *Termitomyces* and by the comb microbiota (Hyodo et al., 2000, 2003; Taprab et al., 2005; Poulsen et al., 2014; Li H. et al., 2017; da Costa and Poulsen, 2018). *Termitomyces* sp. breaks down plant polymers by combining the activity of several carbohydrate-active enzymes (e.g., dextranase [GH49], β-glucuronidase [GH79], xylanase [GH10], xyloglucan [GH16], β-glucosidase [GH3], Cu-dependent lytic polysaccharide monooxygenases [AA9]), oxidizing enzymes (manganese peroxidase [EC 1.11.1.13], dye decolorization peroxidase [EC 1.11.1.19], unspecific peroxygenase [EC 1.11.2.1], laccases [EC 1.10.3.2], and aryl-alcohol oxidases [EC 1.1.3.7]), and hydroquinone-mediated Fenton chemistry (Poulsen et al., 2014; da Costa and Poulsen, 2018; Li H. et al., 2021; Schalk et al., 2021). Metagenomic data reveals that comb microbiota seems also to take place in plant biodegradation, metabolizing hemicelluloses (e.g., xylan) and xylose. This community is dominated by the bacterial phyla Firmicutes (genera *Acetonema* and *Sporomusa*), Bacteroidetes (genus *Alistipes*), Proteobacteria (genera *Pantoea*, *Rahnella*, and *Serratia*), Actinobacteria, and Saccharibacteria. Although mainly gut-derived, the comb bacterial community seems mainly gut-derived, though environmentally acquired bacterial also contribute to the composition (Aylward et al., 2014; Li H. et al., 2016, 2017; Otani et al., 2016). Fungal-bacterial degradation of plant polysaccharides results in an old comb enriched in glucose and oligosaccharides. When old workers feed on the old comb (i.e., the second gut passage), enzymes derived from both the termites and the gut microbiota degrade oligosaccharides and fungal biomass. Workers gut microbiota abundantly presents mannosidases (GH92), xylanases/β-xylosidase (GH43), and β-Galactosidase/β-mannosidase/β-glucuronidase (GH2), related to oligosaccharides metabolism. In this second gut passage, the older gut ultimately produces feces that contain little or no organic material (Ohkuma, 2003; Liu et al., 2013; Poulsen et al., 2014; da Costa et al., 2018; Hu et al., 2019). Workers guts are stated as the central compartment for the symbiosis to operate, as they converge the metabolic potential of each member of the symbiosis (enzymes from the termite, from the gut microbiota, and fungal nodules) toward substrate digestion (Poulsen et al., 2014).

### Hymenoptera-Fungi Mutualisms

#### Structural Fungiculture in Ant-Plants “Domatia”

Ant (Hymenoptera: Formicidae) ecology involves the association with diverse microorganisms, embracing structural, defensive and nutritional symbioses (Figure 2; Moreau, 2020). Fungal cultivation for construction, prey-catching strategy, feeding and defensive purposes evolved in at least 17 plant-ant symbioses. Myrmecophytes (also known as ant-plants) provides hollow structures, named “domatia,” to serve as nesting sites harboring ant colonies. In turn, plant-ants protect the plant host against pathogens and competition, and may further contribute with the myrmecophyte nutrition (Davidson and McKey, 1993; Letourneau, 1998; Rico-Gray and Oliveira, 2007; Defossez et al., 2009; Mayer et al., 2018). Fungal cultivation takes place inside the domatia, on carton structures built from masticated plant and soil materials. There, fungal symbionts from the order Chaetothyriales (Ascomycota) grow while structurally reinforcing carton walls (Maschwitz and Hölldobler, 1970; Kaufmann and Maschwitz, 2006; Leroy et al., 2011; Mayer et al., 2014). Chaetothyriales (black yeasts) are common on both the carton and galleries of ant nests, and in the domatia, though the fungal community seems specific to each environment. Carton community is mainly composed of fungi having monilioid hyphae with thick and dark walls, rendering a dark carton that avoid invasive fungi. Domatia fungi have hyaline or light-brown hyphae with thin walls, assembling a more specific community, where few species co-occur. Complex fungal associations occur in the carton and domatia community, presenting some specificity toward each ant-plant symbiosis (Voglmayr et al., 2011). Chaetothyriales compose an ecologically diverse group of primarily saprotrophic fungi that flexibly change from hyphal to yeast-like growth. These fungi are adaptable to oligotrophic environments, being able to grow in hydrocarbon-rich environments by metabolizing aromatic hydrocarbons as the only carbon source (Prenafeta-Boldú et al., 2006; Satow et al., 2008; Zhao et al., 2010; Voglmayr et al., 2011; Moreno et al., 2019). Plant-ant-fungus interactions were first observed in carton constructions of European *Lasius* (Formicinae) ants (Elliott, 1915; Maschwitz and Hölldobler, 1970; Schlick-Steiner et al., 2008). Ant subgenera *Dendrolasius* and *Chthonolasius* build a composite material from wood and soil particles, bonded together by ascomycete mycelia. Fungal symbionts belong to the Chaetothyriales, Capnodiales, and Venturiaceae, being nourished and managed by the ants (Schlick-Steiner et al., 2008). *Lasius fuliginosus* ants actively manage the carton fungal community through chemical compounds from ants’ glandular secretions that favor symbiont growth while suppressing entomopathogenic fungi. *L. fuliginosus* ants are reported to provide crop-derived sugary solutions for nourishing the fungal symbiont (Maschwitz and Hölldobler, 1970; Brinker et al., 2019).

Plant-ants of the Amazonian genus *Allomerus* (Formicidae: Myrmicinae) cultivate fungus with non-nutritional, but structural purpose, i.e., to reinforce the carton walls of the galleries they build. Associated with the neotropical ant-plant *Hirtella physophora* (Chrysobalanaceae), *Allomerus decemarticulatus* ants uses a galleried structure on the plant stems to hide themselves and to trap and capture prey. The gallery is built by three major steps: (i) the majority of trichomes are cut to clean the stems; (ii) while the trichomes left uncut are used as support, cut ones are bonded together by the mycelium of the ascomycete fungus *Trimmatostroma* sp. (Chaetothyriales); (iii) the fungus grow probably by using plant material as resource, creating a dense mycelial network around gallery openings, that eventually spreads throughout the structure. Hidden inside the gallery, *A. decemarticulatus* workers wait for insect preys with their mandibles open just under the openings, attacking the insect as soon as it lands on the structure (Dejean et al., 2005; Leroy et al., 2011). Furthermore, this system contains a third association, where the fungus growing within the galleries provides nitrogen to the host plant. In old domatia, a dense mycelial network occurs in close proximity to plant cells, a site where the fungus mediate nitrogen uptake by the plant (Leroy et al., 2011, 2017). For cultivating *Trimmatostroma* sp., ants select and process suitable substrates by chewing domatia-extracted material to form pellets. These are applied to trichomes clusters at the gallery foundation, favoring hyphal spreading. Following mycelial establishment, the ants apply prey remains and plant material on the gallery walls, nourishing the fungus (Leroy et al., 2011). Vertical transmission has not been reported, and the ants cultivate a fungal species with few haplotypes of Chaetothyriales (Ascomycota) fungi. Even though fungal spores from diverse species were reported in the galleried structure, the mycelial network is originated from only one symbiont, suggesting mechanisms for suppressing and removing fungal pathogens (Dejean et al., 2005; Lauth et al., 2011; Leroy et al., 2011; Ruiz-González et al., 2011). Since *A. decemarticulatus* workers make a behavioral investment to manipulate, to cultivate, and to clean their symbiont, such ant-fungus association is characterized as a non-nutritional fungiculture, yet to be enzymatically characterized (Lauth et al., 2011).

An analogous hunting strategy was developed by the arboreal ant *Azteca brevis* (Formicidae: Dolichoderinae) inhabiting live stems of *Tetrathylacium macrophyllum* (Salicaceae). *Azteca* ants are also found on *Grias* sp. (Lecythidaceae), *Licania* sp. (Chrysobalanaceae), *Myriocarpa* sp. (Urticaceae), and *Ocotea nicaraguensis* (Lauraceae) myrmecophytes (Longino, 2008; Mayer and Voglmayr, 2009). In natural cavities of *T. macrophyllum* formed due to degeneration of secondary branches, *A. brevis* ants build carton galleries covering the natural openings. The ants collect particles around the tree branches (as bark, cut epiphytes and epiphylls), regurgitating them as pulp to structure lateral pillars, which are later connected by an arch to form a gallery system. Particulate material is connected by a dense mycelial network that become the main component of carton galleries, without invading the host plant. Gallery walls are multi-species networks of at least four Chaetothyriales fungal symbionts, saprotrophs that presumably metabolize the plant particles of the carton as substrate. Carton structure usually presents two types of hyphae: hyaline with thin walls and melanized with thick walls, the latter dominating the mycelial biomass, supposedly for favoring carton stability. Fungal hyphae are nourished and trimmed by the ants to avoid disorganized overgrowth, though the fungus is not used directly for ants nourishment. The resulting galleried carton structure is employed as trap for capturing prey (Mayer and Voglmayr, 2009; Nepel et al., 2014, 2016). In an 8 Mya symbiosis, neotropical *Cecropia* trees (Urticaceae) provide domatia (hollow stem internodes) and glycogen-containing plastids (Müllerian bodies) as nesting site and food, respectively, for *Azteca* ants. The ants, in turn, protect the tree against herbivory, prune it and provide extra nutrients. Fungi are cultivated in *Cecropia* domatia, apparently metabolizing plant defensive volatile organic compounds (VOCs), including monoterpenes (d-limonene, ρ-cymene, and β-phellandrene) and benzothiazole (heterocyclic sulfur-containing compound), potentially harmful to larvae and adult ants. For *Azteca alfari*, *A. coeruleipennis*, *A. constructor*, and *A. xanthochroa*, fungal patches are transferred from the parental colony by the foundress queen. Before layering eggs, queens begin to dig into the domatia spongy parenchima, forming a parenchyma pile (the “foundress patch”). Chaetothyriales fungi are cultivated on these patches, where also the oviposition and larval development takes place. Larvae feed on the fungus while developing, although the ant queen does not do so (Bischof et al., 2013; Gutiérrez-Valencia et al., 2017; Mayer et al., 2018, 2021). Similarly, in the plant-ant symbiosis of *Petalomyrmex phylax* (Formicinae)-*Leonardoxa africana* (Fabaceae), *Tetraponera aethiops* (Pseudomyrmecinae)-*Barteria fistulosa* (Passifloraceae), and *Pseudomyrmex penetrator* (Pseudomyrmecinae)-*Tachigali* sp. (Fabaceae), ant larvae feed on domatia fungi hyphae, transferred to them by adult workers (Blatrix et al., 2012).

#### Advanced Fungiculture of Attine Ants

Fungus-growing (attine) ants cultivate basidiomycete fungi in the families Agaricaceae and Pterulaceae, maintained in sponge-like structures known as fungus gardens (Martin, 1970; Mueller et al., 2005). Based on fungal cultivation practices, fungus-growing ants’ symbioses are categorized in diverse agricultural systems, among which the “lower” and the “higher” fungiculture gather the majority of attine genera (Schultz and Brady, 2008; Branstetter et al., 2017). Excepting ants in the *Apterostigma pilosum* group, that cultivate coral-mushroom fungi in the family Pterulaceae (Munkacsi et al., 2004; Villesen et al., 2004), lower attines cultivate not truly domesticated fungi in the tribe Leucocoprinae (Basidiomycota: Agaricales: Agaricaceae). Leucocoprinae symbionts are able to sustain a free-living existence and were likely acquired multiple times along evolution (Hölldobler and Wilson, 1990; Chapela et al., 1994; Mueller et al., 1998, 2005; Nygaard et al., 2016). Fungal crops are cultivated using flower parts, seeds, wood fragments, plant debris, arthropods feces, and carcasses, similarly to those substrates metabolized by saprotrophic fungi (Harley, 1971; Martin and Martin, 1971; Deacon et al., 2006; De Fine Licht and Boomsma, 2010). The lignocellulolytic activity of leucocoprinaceous crops relies on cellulases, hemicellulases, and lignin-modifying enzymes (De Fine Licht et al., 2010; Nygaard et al., 2016), sharing an overall enzymatic profile with free-living saprotrophic agaricaceous (Leger et al., 1997; De Fine Licht et al., 2010). Indeed, the fungal crop of lower attines in the genus *Cyphomyrmex* codifies for more polysaccharides and lignin-degrading genes than free-living relatives, suggesting the recruitment of flexible decomposers by early farmers (Nygaard et al., 2016). Without apparent fungal adaptations for living in symbiosis, lower attines fungiculture is thought as a litter-decomposing saprotrophic system, extended by ant management and provisioning (Vo et al., 2009; De Fine Licht et al., 2010, 2014). Such microbial environment seems shaped by the ants in some extent, possibly by filtering-out unwanted microorganisms by grooming and exchanging bacteria across the garden. Differing from the soil surroundings, the bacterial community of *Mycocepurus smithii* is dominated by *Lactobacillus* and *Pantoea.* Differing from the overall pattern presented by other lower and higher attini (and even other fungus-growing insects), the bacterial community of *Mycocepurus goeldii* dominated by *Pseudomonas, Dysgonomonas, Bacteroides, Enterobacter, Parabacteroides, Prevotella, Comamonas*, and *Burkholderia* (Kellner et al., 2015; Barcoto et al., 2020).

On the other hand, higher attines fungal crops have extensive nutritional and morphological adaptations for a symbiotic life (Chapela et al., 1994; Schultz and Brady, 2008; De Fine Licht et al., 2014; Nygaard et al., 2016). Such adaptations possibly blocked gene flow between free-living relatives and the cultivar, which seems unable to sustain a free-living existence (Mueller et al., 1998, 2005; Schultz and Brady, 2008; De Fine Licht et al., 2014). All higher attines cultivate gongylidia-bearing fungi in the genus *Leucoagaricus* (Agaricaceae: Basidiomycota). Gongylidia are swollen hyphae containing vacuoles filled with essential aminoacids, lipids, free sugars, polysaccharides, and plant-degrading enzymes (Quinlan and Cherrett, 1979; De Fine Licht et al., 2014; Aylward et al., 2015). Ants are attracted to the lipids in these specialized structures that compliment adult workers diet and are the sole food source for the ant queen and larvae (Quinlan and Cherrett, 1979; De Fine Licht et al., 2010, 2014; Khadempour et al., 2021). Leaf-cutting ants, the most derived higher attines, evolved a sophisticated shift in diet composition and substrate preparation, as they actively cut fresh leaves, flower petals, and fruits to cultivate the fully and highly domesticated *Leucoagaricus gongylophorus* (De Fine Licht and Boomsma, 2010; De Fine Licht et al., 2010, 2014). Leaf-cutting ant colonies function as generalist herbivores, supported by the high metabolic activity of their fungal crop, adaptable to substrate composition and recalcitrance (Bucher et al., 2004; Kooij et al., 2011; Shik et al., 2014; Khadempour et al., 2016).

Fresh foraged material is brought inside the ant colony, cleaned, and fragmented by licking, masticating, and removing the wax layer (Hölldobler and Wilson, 1990; Currie and Stuart, 2001; Garcia et al., 2005). Ant fecal fluid droplets added to the pulped substrate contribute for treating it with gongylidia-produced proteases, hemicellulases, pectinases, laccases, and oxidoreductases. In a complementary physiological process, these enzymes pass unharmed through the ant gut after being ingested, ultimately aiding the degradation of plant biomass (Martin and Martin, 1971; Boyd and Martin, 1975; Rønhede et al., 2004; Schiøtt et al., 2010; De Fine Licht et al., 2013; Kooij et al., 2014, 2016; Aylward et al., 2015; Schiøtt and Boomsma, 2021). Oxidoreductases such as laccase, 4-carboxymuconolactone decarboxylase, copper radical oxidase, glyoxal oxidase, galactose oxidase, isoamyl alcohol oxidase, glucose-methanol-choline (GCM) oxidoreductases, aryl-alcohol oxidases are abundant in the fecal fluid of *Acromyrmex echinatior*. Out of these, GCM oxidoreductases seem to produce high quantities of H_2_O_2_, which interact with chemicals of pulped leaves *via* inorganic Fenton reactions, producing hydroxyl radicals that may assist lignocellulose breakdown (Schiøtt and Boomsma, 2021). Acting as an external herbivorous gut, these complex gardens present defined strata (Figure 2): (i) the gray-green top layer where fresh leaves are incorporated; (ii) the white middle layer having gongylidia clusters; (iii) the gray-brown bottom layer enriched in non-degraded recalcitrant plant polymers (Martin, 1970; Mueller et al., 2005; Schiøtt et al., 2008, 2010; Moller et al., 2011; Aylward et al., 2013; Grell et al., 2013; Lange and Grell, 2014; White et al., 2014). Despite having lost an ancestral ligninase domain, *L. gongylophorus* encodes for laccases, glyoxal oxidases, cellulases (GH6, GH7, GH9), hemicellulases and pectinases (CE5, CE8, GH15, GH28, and PL1; Aylward et al., 2013; Nygaard et al., 2016). The garden microbiota, dominated by *Enterobacter*, *Klebsiella, Pantoea, Pseudomonas*, and *Serratia*, encode for metabolic pathways that could compliment the fungal metabolism (Scott et al., 2010; Suen et al., 2010; Aylward et al., 2012; Barcoto et al., 2020; Francoeur et al., 2020; Khadempour et al., 2020).

Plant biomass turnover in the fungus garden takes about six weeks, targeting specific plant components in each stratum (Moller et al., 2011; Somera et al., 2015; Shik et al., 2018; Francoeur et al., 2020; Caraballo-Rodríguez et al., 2021). At the top, pectinases and hemicellulases likely allow the access of fungal hyphae to the main nutritional target, i.e., proteins and starch within plant cells (Moller et al., 2011; Aylward et al., 2013; Grell et al., 2013). Consequently, arabinose, mannose, xylose, glucose, and polyols are accumulated at the top, supporting an initial microbial growth (Somera et al., 2015). Laccases are also abundant at the top garden, thought to be produced in gongylidia and vectored *via* the fecal fluid to detoxify plant phenolic compounds (Aylward et al., 2013; De Fine Licht et al., 2013; Grell et al., 2013). High metabolic activity and abundance of gongylidia characterize the middle layer, where the fungal crop quickly integrates carbon and nitrogen into edible gongylidia (Shik et al., 2018). More recalcitrant plant polymers are metabolized at the bottom layer that characteristically express cellulases, hemicellulases, pectinolytic enzymes, lipases, and cutinases. Low glucose concentration at the bottom may stimulate cellulase activity, which could sustain the fungal growth, though not contributing to the ants’ nutrition (Schiøtt et al., 2008; Aylward et al., 2013; Grell et al., 2013). Old garden material containing recalcitrant polymers is discarded by the workers, and the degradation continues in the debris pile. There, some members of the microbiota dominated by γ-Proteobacteria and Bacteroidetes possibly assist cellulose degradation (Bot et al., 2001; Scott et al., 2010; Moller et al., 2011; Lewin et al., 2016). Defensive metabolites, like polyunsaturated fatty acids and phenolic derivatives, are also transformed along with plant degradation in the garden and waste piles (Caraballo-Rodríguez et al., 2021).

#### Woodwasps “External Rumen”

Adult and larvae of woodwasps (Hymenoptera: Siricidae and Xiphydriidae) rely on fungal symbionts to convert the wood of dying and dead standing trees into more labile compounds. Tree colonization starts when adult females excavate into the xylem tissues to oviposit, constructing galleries that are inoculated with fungal spores (Morgan, 1968; Kukor and Martin, 1983; Thompson et al., 2014). The symbiosis association between *Sirex noctilio* (Hymenoptera: Siricidae) and the white-rot fungi *Amylostereum areolatum* (Basidiomycota: Corticiaceae) is highly aggressive and invasive, causing the death of living trees under mass attack (Figure 2; Morgan, 1968; Coutts, 1969; Slippers et al., 2015). Monoterpene hydrocarbons volatiles, as α- and β-pinene, are emitted by stressed hosts attracting mated *S. noctilio* females. In pine hosts already infected, *A. areolatum*-derived volatiles could be even stronger attractants, hypothetically indicating a suitable substrate for gallery formation (Fernández Ajó et al., 2015). Carrying the fungal symbiont in the mycangia, *S. noctilio* females perforates test bores, oviposing in the most suitable according to moisture content and resin pressure. While laying eggs in the xylem of *Pinus* spp., *S. noctilio* female fill the tunnels with phytotoxic mucus (“venom mucus”) and inoculate *A. areolatum* spores, causing host physiological stress that ultimately favors egg eclosion and larval development (Madden, 1977; Slippers et al., 2011). Venom mucus is produced in the venom glands, expressing diverse host-damaging toxins, such as glycopeptide noctilisin, acid phosphatase proteins, disintegrins and metalloproteinases. These peptides induce pine defensive responses, potentially damaging several physiological process, including needle wilt, chlorosis, abscission, carbohydrates translocation and phloem collapse (Madden, 1977; Bordeaux et al., 2014; Wang et al., 2016). *A. areolatum* grows within the tunnels, decomposing wood by secreting lignocellulolytic enzymes, including families of cellulases and xylanases (GH3 and GH5), glucose-methanol-choline (GMC) family of oxidoreductases (AA3), and laccase (AA1), which are abundantly encoded in the fungal genome (Thompson et al., 2014; Fu et al., 2020, 2022).

*Sirex noctilio* larval development depends on *A. areolatum* enzymatic activity, and their nutritional interaction does not seem strictly based on mycophagy. Taking advantage of an “external rumen,” *S. noctilio* larva consume plant nutrients derived from fungal pre-digestion, as starch and simpler polysaccharides. Larvae have sterol molecules supplied by pine phytosterols instead of *A. areolatum* ergosterol, further suggesting that pre-processed wood is their main nutritional resource. Larvae forage around the border of fungal growth, where enzymatic conversion of lignocellulose is more active, possibly providing higher amounts of nutrients. Larvae also ingest fungal cellulases and xylanases that facilitate gut metabolism of polysaccharides. Their specialized asymmetric mandibles press xylem fragments to extract pre-digested fluids, then using a sulcus on the left mandible to drain liquids for the oral cavity. Instead of being ingested, xylem fragments are expelled from the oral cavity, and few xylem particles were detected in larvae gut (Kukor and Martin, 1983; Thompson et al., 2013, 2014; Li J. et al., 2021). Gut microbiota composition changes along larval growth and development, differing from those of adult gut and processed xylem. While the larval gut is enriched in *Pseudomonas*, the adult male gut is dominated by *Ralstonia*, the adult female gut has high abundance of *Acinetobacter*, and the processed xylem fragments are enriched in *Methylobacterium* (Li J. et al., 2021). Cellulolytic activity of the larval gut isolates *Streptomyces* and *Pantoea* points that bacteria and *A. areolatum* could complimentary degrade lignocelulose (Adams et al., 2011b). The fungal community is constantly dominated by the genera *Amylostereum, Tremella*, and *Malassezia* along *S. noctilio* development. *Amylostereum* seems particularly abundant in larvae gut, suggesting, together with putative fungal-degrading salivary gland secretions, that mycophagy contribute to larvae nutrition in some extent (Talbot, 1977; Li J. et al., 2021).

A similar, tough less understood interaction, evolved between the woodwasp *Xiphydria* spp. (Hymenoptera: Xiphydriidae) and the xylariaceous *Daldinia* spp. and *Entonaema cinnabarina* (Xylariales: Ascomycota). Such associations seem species-specific since the *X. longicollis* mycangia may be inhabited by *D. childiae, D. decipiens* and *E. cinnabarina* while *X. prolongata* mycangia was reported to carry *D. childiae and E. cinnabarina*. *D. decipiens* predominated in the *X. picta* and *X. camelus* mycangia, though the latter also presented *D. childiae* and *D. petriniae* symbionts (Šrùtka et al., 2007; Pažoutová et al., 2010). *Xiphydria*-xylariaceous fungi symbiosis appear to take advantage of fungal lignocellulolytic capacity, as suggested by the endophytic *Daldinia eschscholtzii* EC12, whose genome encodes for diverse plant-degrading enzymes targeting cellulose, hemicellulose (xylan, xyloglucan, pectin), and lignin (Chan et al., 2015; Hori et al., 2020). Indeed, *D. decipiens* oita isolated from *X. albopicta* metabolized glucose, cellulose, xylan, mannan, pectin, poplar, and larch by secreting enzymes related to cellulose and hemicellulose degradation. *D. decipiens* oita regulate its enzymatic response to the lignocellulosic content of the substrate. Cellulose, poplar, and larch induced the activity of cellobiohydrolase (CBH)/endo-glucanase (GH6 and GH7), endo-xylanase (GH11), acetyl xylan esterase (CE1), and 4-O-methyl-glucuronyl methylesterase (CE15), while pectin induced rhamnogalacturonan endolyases (PL4), and xylan induced various CAZymes, including arabinan endo-α-1,5-arabinosidase (GH43), CE1, and PL4. Some carbon sources also induced the secretion of gluco-oligosaccharide oxidase (AA7), glyoxal oxidase (AA5), and GMC oxidoreductase (AA3). Since laccases or other AA1 peroxidases were not detected in the proteome, instead of using oxidative lignin degradation, *D. decipiens* oita could employ the LPMO system (Hori et al., 2020).

#### Pre-digestion and Steroid Supplementation for Meliponini Bees

Stingless bees (Hymenoptera: Apidae: Meliponini) nests are perennial and commonly built in cavities, where they store food as nectar and pollen. Diverse symbiotic microorganisms inhabiting Meliponini hives participate in physiological processes required for converting pollen and nectar by secreting enzymes, fermenting polysaccharides, producing organic acids (Menezes et al., 2013; de Paula et al., 2021). Besides producing antimicrobial compounds, bacteria isolated from *Heterotrigona itama* present cellulolytic, proteolytic, and lipolytic activity. Such enzymes, mainly secreted by *B. cereus* HD1, *B. amyloliquefaciens* PD9, *B. safensis* BD9, and *B. subtilis* BD3, might participate in degrading complex molecules during bee digestion (Ngalimat et al., 2019). As soon as pollen is stored, fermentative processes are initiated, increasing moisture and lactic acid content as fermentation progress. By fermenting stored nectar and pollen, microbial symbionts aid in preserving food and forming nest products. *Scaptotrigona depilis* bees in fact preferred fermented pollen rather than fresh pollen (Menezes et al., 2013; Vollet-Neto et al., 2017; Ngalimat et al., 2019). Yeasts in the genera *Starmerella* are commonly associated with Meliponini, fermenting nectar together with bacteria (Daniel et al., 2013; Santos et al., 2018; Costa Neto and Morais, 2020). Fungus growing on the walls of brood cells of the bees *S. depilis, Tetragona clavipes*, and *Melipona favolineata* suggest bee-fungi symbioses involving fungal steroid supplementation. Insects require ecdysteroids for metamorphosing, since these sterol-derived molting hormones trigger metabolic cascades that transform immatures into adults. As insects are not able to synthesize sterols *de novo*, they rely on steroid precursors from the diet, which in some Meliponini include fungal eating (Dubrovsky, 2005; Menezes et al., 2013; Lavrynenko et al., 2015). On the walls of *S. depilis* brood chamber grows *Zygosaccharomyces* sp. (Ascomycota: Saccharomycetales), favored by high carbohydrate content and low pH characteristic of chamber environment. *Zygosaccharomyces* sp. forms a pseudomycelium while growing, supposedly facilitating larvae consumption. When larvae feed on fungi, fungal ergosterol may enter *S. depilis* pathways to synthesize ecdysteroids, providing nutritional and hormonal support for larvae morphogenesis and molting (Paludo et al., 2018). Such symbiosis is hypothesized to be influenced by other microorganisms frequently found in Melipolini hives, as *Monascus* spp. (Ascomycota: Eurotiales) from *S. depilis* and *Melipona scutellaris*, and *Candida* spp. (Ascomycota: Saccharomycetales) from diverse stingless bees. In *in vitro* co-culture assays, *Candida* spp. produce VOCs (as ethanol and isoamyl alcohol) that stimulate *Zygosaccharomyces* sp. to grow, and *Monascus ruber* produces monascin and lovastatin, having antagonistic effects on *Candida* spp. and *Zygosaccharomyces* sp., respectively. In this scenario, larval development would be intricately dependent on microbial interactions occurring on brood cells (Menezes et al., 2013; Paludo et al., 2018; de Paula et al., 2021).

### Coleoptera-Fungi Mutualisms

#### Yeast Fungiculture as Nutritional Support for Larval Development

For obtaining nutritional resources from poor-nutrient wood, ship timber beetles (Coleoptera: Lymexylidae) cultivate *Alloascoidea* (Ascomycota: Saccharomycetales) symbionts, though to metabolize wood component and make labile sugars available to the beetle (Figure 2). Lymexylid beetles *Elateroides dermestoides* and *E. flabellicornis* carry the symbiont fungus within mycangia, inoculating the wood with fungi during oviposition. In freshly dead wood, *E. dermestoides* females oviposit forming eggs clusters, which are covered with a secretion containing *Alloascoidea hylecoeti.* The symbiont inoculated on the egg surface is consumed as soon as *E. dermestoides* larvae hatch, and then while excavating the wood, feeding on *A. hylecoeti* growing on the gallery walls. Since *A. hylecoeti* metabolize cellobiose, rhamnose, xylose, and arabinose, its role in the symbiosis could involve providing wood-derived sugars to support larval development (Francke-Grosmann, 1967; de Hoog and Th Smith, 2011). *E. flabellicornis* females also carry *Alloascoidea* sp. in the mycangia, along with *Ambrosiozyma llanquihuensis*, *Ambrosiozyma* sp., *Cyberlindnera* sp., and *Saccharomycopsis* sp. Although all fungal symbionts assimilated glucose and xylose, galactose and galacturonic acid were metabolized only by *Saccharomycopsis* sp., xylan only by *Alloascoidea* sp. and *Cyberlindnera* sp., and cellobiose only by *Saccharomycopsis* sp. and *Cyberlindnera* sp. Association with diverse fungal symbionts with different metabolic capacity may diversify food sources available for *E. flabellicornis* larvae (Toki, 2021). Females of the lizard beetle *Doubledaya bucculenta* (Coleoptera: Erotylidae: Languriinae) oviposit inside the internodes of recently dead *Pleioblastus* and *Semiarundinaria* bamboo culms (Figure 2). They excavate bamboo internodes until reaching the cavity, where one egg is deposited along with the symbiotic yeast *Wickerhamomyces anomalus* (Ascomycota: Saccharomycetales). The yeast is carried from the parental garden inside the mycangia, being inoculated both on the egg surface and internode inner walls. *W. anomalus* grows by assimilating bamboo-derived mono- and polysaccharides, in particular free sugars, as glucose and fructose. Only one *D. bucculenta* larvae develops per internode, inoculating, spreading, maintaining, and feeding on the yeast garden (Toki et al., 2012; Toki and Togashi, 2013; Toki and Aoki, 2021).

#### Detoxification and Nutritional Supplementation for Bark Beetles

Fungus farming in Curculionidae weevils evolved independently at least 13 times in the subfamily Scolytinae and one time in the subfamily Platypodinae (Jordal and Cognato, 2012; Biedermann and Vega, 2020). This polyphyletic assemblage of ecologically similar weevils includes ambrosia beetles, derived from phloeomycetophagous bark beetles (Jordal and Kambestad, 2014; Kasson et al., 2016; Biedermann and Vega, 2020; Peris et al., 2021). Bark beetles (Coleoptera: Curculionidae: Scolytinae) are woodborer weevils that breed in the phloem (i.e., inner bark) of woody plants. This diverse and speciose group of beetles present diversified feeding strategies that include phloeophagy (when larvae feed on phloem) and mycophoeophagy (when larvae feed on phloem associated with fungi). Phloem is more nutritious than bark and wood, yet this tissue has low concentrations of nitrogen and sterols, for which several bark beetles species take advantage of the nitrogen-accumulating capacity and ergosterol content of fungi. Conifer-colonizers bark beetles benefit from fungal detoxification of tree defensive compounds and pheromones (Paine et al., 1997; Six, 2012, 2013; Kirkendall et al., 2015; Raffa et al., 2015). Even though the association with fungi is widespread among bark beetles, because their major food item is phloem they are not considered truly fungal farmers (Harrington, 2005; Six, 2012; Hulcr and Stelinski, 2017). Bark beetle reproductive strategies vary according to the species, and for those associated with fungal partners, narrow galleries are excavated in the bark of the host tree by either the female, the male, or both. Along with gallery building, associated fungi are inoculated, growing by metabolizing phloem and wood components. The female oviposit along the gallery or in an excavated chamber, and after ecloding, larvae develop by consuming phloem tissue (associated or not with fungi). This consequently extend the galleries length, irradiating as far as 10–15 cm from the original gallery. At the end of the gallery, the larvae may build a chamber for pupation, where fungal sporulation is essential for its dispersion to a new host tree. Young adults emerging from pupae may require a maturation feeding before emerging, supposedly consuming fungi and bark. Mature adults then emerge from pupae chamber carrying fungal spores (Harrington, 2005; Kirkendall et al., 2015; Raffa et al., 2015).

Bark beetle-fungi associations range from highly specific (where the fungus is associated with one beetle species) to non-specific (where the fungus may be associated with diverse beetle species). Such associations also vary from facultative to obligatory, and whether the beetle present mycangia for carrying fungal spores is an indicative of high mutual dependency (Six, 2012). Mycangial bark beetle species seem dependent on the fungal partner for nutritional supplementation. For instance, larval development of the mycophloeophagous *Dendroctonus brevicomis* relies on the associated fungus to transport and concentrate nitrogen and phosphorous from xylem and phloem, enriching its diet with these elements (Six and Elser, 2019). Fungal partners could supply the beetles’ sterols requirements, and indeed ophiostomatoid symbionts of *D. ponderosae* and *Dendroctonus rufipennis* present high ergosterol content (Bentz and Six, 2006; Six, 2013). Dependency seems also related to the niche occupied by the insect and colonization behaviors. Aggressive colonizers, as *D. brevicomis* and *D. ponderosae*, are mycangial bark beetles that obligatory depend on two specific bionecrotroph fungi (i.e., those that overcome tree defenses and invade living tissues) for nutritional supplementation. Aggressive secondaries, as *I. typographus* and *D. rufipennis*, have variable associations with bionecrotroph fungi, relying in phloem as main nutritional source. When consistently associated with fungi, aggressive secondaries may present simple pits as mycangia, though mycangia is absent in those species without a fungal symbiont. Non-aggressive secondaries are loosely associated with non-specific commensal fungi, and fungal partners are lacking in parasite species of bark beetles (Six, 2020).

The most commonly associated fungi include ophiostomatoid fungi *Ophiostoma, Ceratocystiopsis, Grosmannia* (Ascomycota: Ophiostomatales) and *Ceratocystis* (Ascomycota: Microascales). Most bark beetles appear to be associated with two or more fungal symbionts, which are mainly bionecrotrophs ultimately facilitating tree colonization (Kirisits, 2004; Zipfel et al., 2006; Six, 2012, 2013). *I. typographus* attacks to stressed Norway spruces (*Picea abies*) involve in overcoming the conifers’ defensive oleoresin, composed of monoterpenenoids, sesquiterpenenoids, and diterpenenoids. *Endoconidiophora polonica* (previously *Ceratocystis polonica*; Ascomycota: Microascales), *Grosmannia penicillata*, and *Grosmannia europhioides*, blue-stain fungi commonly associated with *I. typographus*, convert terpenoids (as limonene), stilbenes and flavonoids into ring-opened products. Possibly as the first step of the β-ketoadipate pathway for metabolizing aromatic compounds, *E. polonica* converts stilbenes into stilbene dimers, aglycones, and ring-opened lactones. This may involve cleaving 3,4-hydroxy rings *via* catechol dioxygenases enzymes (DiGuistini et al., 2011; Hammerbacher et al., 2013; de Beer et al., 2014; Wang et al., 2014; Wadke et al., 2016; Zhao et al., 2018). In the heterogeneous nutritional landscape provided by an infected conifer, context-dependent interactions between fungal symbionts may determine the colonization success. Associated with the bark beetle *Dendroctonus ponderosae, Grosmannia clavigera, Ophiostoma montium*, and *Leptographium longiclavatum* emit fungal VOCs acting not only as semiochemicals, but also as carbon source metabolized by fungi growing in nutrient-poor substrates, constituting a cross-feeding network (Cale et al., 2016).

Symbiont dynamics is also modulated by the bacterial community, that could both stimulate the growth of symbiont fungi when tree defensive compounds (in special α-pinene) are present, or inhibit fungi when α-pinene is absent. The gallery bacterial community, where *Serratia, Pseudomonas, Stenotrophomonas*, and *Erwinia* predominate, encodes for pathways related to degradation of mono- and diterpenes (Cardoza et al., 2006; Scott et al., 2008; Adams et al., 2009, 2011a,^2013^; Raffa, 2014; Zhou et al., 2016). Some of these strains utilized monoterpenes as carbon source *in vitro*, in special *Pseudomonas mandelii*, *Pseudomonas migulae*, *Rahnella aquatilis* and *Serratia marcescens* (Adams et al., 2013; Boone et al., 2013). Also, a naringenin-degrading bacterial community associated with *Dendroctonus valens* externally detoxify such phenolic compound (Cheng et al., 2018). Bacterial species associated with the bark beetles symbioses tolerate and metabolize terpenes and phenolic compounds, inhibit antagonistic fungi, possibly provide carbon and nitrogen to the host beetle, and encode pathways for degrading lignocellulose (Morales-Jiménez et al., 2009, 2012, 2013; Menéndez et al., 2015; Fabryová et al., 2018; García-Fraile, 2018; Xu et al., 2019; Peral-Aranega et al., 2020; Saati-Santamaría et al., 2021). *Pseudomonas* spp., in particular, have been consistently found in diverse bark beetle species, in diverse niches, and throughout the beetle lifecycle, hypothesized as a consistent member of the beetle-fungal symbiosis (Saati-Santamaría et al., 2021).

#### Fungiculture of Ambrosia Beetles

Xylomycetophagous ambrosia beetles hypothetically derived from bark beetles (Figure 2; Farrell et al., 2001). “Ambrosia” refers to the fungal biomass on which the ambrosia beetles (Coleoptera: Curculionidae: Scolytinae and Platypodinae) larvae obligatory and adults eventually feed (Batra, 1985). Ambrosia fungal symbionts rely on their beetle host for dispersal and maintenance while nourishing the insect host (Biedermann and Taborsky, 2011; Hulcr and Stelinski, 2017). Galleries are often dominated by one fungal cultivar (the ambrosia fungus) that shares this environmental niche with other symbionts (auxiliary microbes). Though the mycangial microbiota presents beetle–fungi congruencies, the galleries’ microbial community seems more specific to the tree host than the beetle host (Batra, 1966, 1985; Harrington, 2005; Bracewell and Six, 2015; Kostovcik et al., 2015; Skelton et al., 2019). Filamentous *Raffaelea* (Ascomycota, Ophiostomatales) and *Ambrosiella* species (Ascomycota, Microascales) are widely documented obligatory fungal mutualists. In addition, *Meredithiella*, and *Phialophoropsis* (Ascomycota, Microascales), Ambrosia *Fusarium* Clade and *Geosmithia* (Ascomycota, Hypocreales), Russulales (Basidiomycota), and the yeast genus *Ambrosiozyma* (Saccharomycetales) are important symbionts (van der Walt, 1972; Harrington et al., 2010; Kolaøík and Kirkendall, 2010; Hulcr and Stelinski, 2017; Aoki et al., 2019; Veselská et al., 2019).

Adults vector fungal conidia between trees, carrying them in specialized and dynamic structures known as mycangia (Kirkendall et al., 2015; Kostovcik et al., 2015; Li et al., 2018, 2019; Spahr et al., 2020; Joseph and Keyhani, 2021). Such structures vary in morphological complexity, from simple pits to elaborate pockets lined with glandular tissue that nourish associated fungi (Batra, 1985; Li et al., 2018). Mycangial microbiomes vary according to beetle species, often dominated by *Raffaelea* and *Ambrosiella* (both were not reported as free-living genera), and also including Pseudomonadales and Burkholderiales bacterial members (Hulcr et al., 2012; Mayers et al., 2015; Wingfield et al., 2017; Carrillo et al., 2019; Ibarra-Juarez et al., 2020). Foundress females are attracted to stressed, dying, or freshly dead trees probably by recognizing emissions of ethanol and/or other volatiles from physiologically stressed host tissues (Graham, 1968; Kimmerer and Kozlowski, 1982). Ambrosia fungus gardens are formed while the foundress female bores tunnel systems in the xylem and inoculates massive amounts of cultivar conidia in the galleries walls. When larvae emerge, while they feed on the fungus they also damage sapwood elements. This facilitates fungal mycelial growth and contributes to the crop dominance in galleries (Batra, 1967, 1985; French and Roeper, 1972; Beaver, 1989; Biedermann et al., 2012). Fungal growth is benefited both by beetles tending the garden and by tree-derived ethanol (Francke-Grosmann, 1967; Harrington, 2005; Hulcr and Stelinski, 2017; Ranger et al., 2018; Lehenberger et al., 2021a). *Ambrosiella* and *Raffaelea* biomass increase when growing in an ethanol-enriched medium, indicating these isolates can tolerate, produce, and detoxify ethanol *via* alcohol dehydrogenase activity (Ranger et al., 2018; Lehenberger et al., 2021a).

Throughout its lifecycle, an ambrosia beetle feeds on primary and secondary fungal symbionts, whose hyphae branch into xylem and phloem, producing spores on gallery walls. When tending beetles are present, the crop mycelium tends to produce chains of ambrosia cells or propagules (Batra, 1966, 1985). As sapwood constitute a nutrient-poor substrate, ambrosia fungal hyphae are thought to transport essential nutrients from the xylem, concentrating them in asexual fruiting structures forming the ambrosia layers. Calcium, nitrogen, phosphorus, potassium, magnesium, and sulfur are made available to ambrosia beetles by consuming such nutritious fruiting structures (Fengel and Wegener, 2011; Filipiak and Weiner, 2014; Filipiak et al., 2016; Lehenberger et al., 2021b). In addition, nutrients could be recycled from beetles’ feces through the crop and microbiota metabolic activity (Batra, 1963, 1967; Lehenberger et al., 2021b). Nitrogen derived from urea and uric acid in beetles feces could favor fungal growth, in turn providing essential amino acids, vitamins, and sterols to the beetles (Batra, 1967; Norris and Baker, 1969; Kok et al., 1970; De Fine Licht and Biedermann, 2012; Lehenberger et al., 2021b). The metabolic profile of ambrosia fungi is mainly determined by their phylogenetic origin, likely optimized for the environmental niches they occupy. Rather than metabolically convergent, each ambrosia symbiosis could be functionally different and have a particular ecology (Huang et al., 2019, 2020).

Ambrosia fungi are not efficient cellulose decomposers instead, they target hemicelluloses that constitute the xylem ray-parenchyma cells, as well as simple sugars (De Fine Licht and Biedermann, 2012). Aside from this pattern are the cellulolytic *Phialophoropsis* (Microascales: Ceratocystidaceae) cultivated by *Trypodendron* beetle species, and the wood-decaying *Flavodon ambrosius* (Basidiomycota: Polyporales) cultivated by *Ambrosiodmus* and *Ambrosiophilus* beetle species (Kasson et al., 2016; Li Y. et al., 2017; Lehenberger et al., 2019). *Flavodon ambrosius* decomposing activity seems to be based on polyphenol oxidase reactions and tannic acid detoxification (Kasson et al., 2016). Similarly, polyphenol oxidase activity was reported for the *Fusarium* sp. AF-4 symbiont of *Euwallacea validus*, and the *Fusarium* sp. AF-9 symbiont of *Xyleborus ferrugineus* can grow using lignin (Kasson et al., 2013). In addition, a core microbiota comprised of *Stenotrophomonas, Enterobacter, Burkholderia*, and *Ochrobactrum* could assist wood and fungal biomass degradation. In galleries of *X. affinis*, microbial communities are temporally and spatially dynamic, possibly shaped by substrate decomposition. While initial stages of gallery development (up to 15 days) are accompanied by bacteria encoding pathways for metabolizing cellulose, hemicellulose, mannan, and rhamnose, the later stages (after 30 days) present more abundant lignin-degraders. However, these patterns may not represent the gallery microbial dynamics of every ambrosia beetles’ symbioses (Ibarra-Juarez et al., 2020). Maintaining a symbiotic association with efficient wood-degrading microbes could enlarge the nutrient availability for the beetle, sustaining the development of complex social structures and overlap of generation (Kasson et al., 2016; Li Y. et al., 2017).

## What Biotechnology Could Learn From Insect Fungiculture?

Microbial catabolic flexibility and adaptability ultimately delimitate the range of forgeable substrates and with that, geographic distribution and ecosystem impact of insect fungiculture. Mediating the use of plant material for structural and nutritional purposes, microorganisms associated with insect fungiculture are the main responsible for degrading and detoxifying recalcitrant and defensive components. Although yet to be detailed, these microbial communities appear to integrate inter-kingdom biodegradation networks, where the labor of degrading and detoxifying plant components is shared between microbial members (Poulsen et al., 2014; Zhang et al., 2018; Barcoto et al., 2020; Francoeur et al., 2020; Ibarra-Juarez et al., 2020). They could complete the required enzymatic pathway in a multipartite configuration, coordinating their respective enzymatic repertoire in tandem to access nutritional resources (White et al., 2014; West and Cooper, 2016; Zhang et al., 2016; Tsoi et al., 2018; Giri et al., 2019). Such synergistic interactions could render a diversified, redundant, and resilient community, expanding the spectrum of substrates and promoting metabolic flexibility (Zhang et al., 2018). We consider there is much to learn from these symbioses, in special from the community-level degradation of recalcitrant biomass and defensive metabolites. Knowledge on the most efficient natural degradation systems could lead to identifying microbial degraders, enzymatic pathways, and metabolic interactions favoring biodegradation. Such tools would allow developing and optimizing microbial consortia for microbial-derived bioprocesses, as recycling and upcycling of agroindustrial lignocellulosic residues, plastic waste, and other pollutants (Ohkuma et al., 2001; Jaiswal et al., 2020; Viljakainen and Hug, 2021). Several plant-degrading microorganisms were already identified in insect fungiculture by culture-dependent and –independent techniques, though their application in bioenergy and bioremediation waits for being fully investigated. Their biotechnological potential may be exemplified by the relatively simple cellulolytic cocktails secreted by *Streptomyces* strains isolated from *D. ponderosae, D. frontalis*, and *S. noctilio*, that could inform on how to simplify processes in lignocellulosic biorefineries (Book et al., 2014). Also, bacterial strains in the genera *Curtobacterium, Erwinia, Pantoea, Pseudomonas, Rahnella, Staphylococcus*, and *Yersinia* were isolated from bark beetles *Cryphalus piceae, I. typographus* and *Pityophthorus pityophthorus* (larvae and adults). Several of these strains were able to hydrolyze plant components *via* cellulases, xylanases, and amylases *in vitro*. The genome of *Pseudomonas* and *Rahnella* strains indicate cellulose degradation as an outcome of coordinated enzymatic activity, since these strains encoded cellulolytic pathways only partially. In addition, some of the bacterial isolates degraded azo-dyes, such as Toluidine Blue, Remazol Brilliant Blue R, Eriochrome Black T, Congo Red, Amido black, and Malachite green (Fabryová et al., 2018).

In order to take advantage of microbial flexible and adaptable metabolism, the characterization of both the taxa and the functional profile of the microbiota is required. Designing strategies for biotechnology becomes possible when knowing which microbes compose the community, how and when they interact to each other, and what are the outcomes of their biochemical communication. Otherwise, the consortia activity could be ineffective and/or result in ecotoxicological outcomes (Cavaliere et al., 2017; Saraiva et al., 2021). For instance, the bacterial community inhabiting dump piles of leaf-cutting ant *Atta colombica* seem to rely on a multipartite cellulolytic activity. *Acidovorax* dominated cellulolytic communities in enrichment experiments, though not growing using cellulose as the sole carbon source. Hence, cellulolytic microorganisms may require nutrients or stimuli from other community members, implicating that microbial networks would be more effective for bioenergy applications (Lewin et al., 2016; Zhang et al., 2018). Microbial interactions fluctuate along time and space, adapting to the composition of substrates and reconfiguring as novel niches are created during substrate degradation (Cavaliere et al., 2017). Exploring fungiculture ecosystem functioning could inform on which microbes are interacting in each stage of plant degradation, through which metabolic pathways they do so, as well as which condition renders synergistic interactions. In the following sections, we point to the knowledge that investigating the microbial found associated with insect fungiculture could provide, as well as biotechnological strategies they could inspire.

### Lesson 1: Who Is There and What Are They Doing?

**FIGURE 3:**
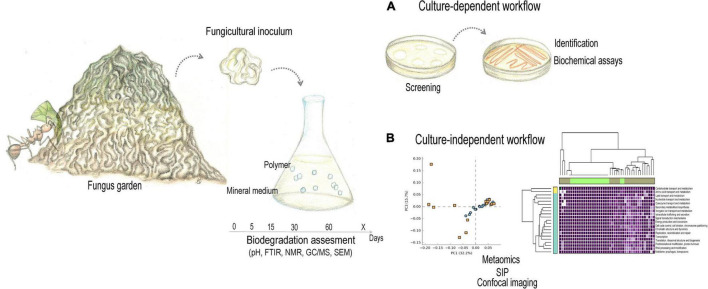
Learning from symbioses. To take advantage of microbial flexible and adaptable metabolism, the characterization of both the taxa and the functional profile of the microbiota is required. Designing strategies for biotechnology becomes possible when knowing which microbes compose the community, how and when they interact to each other, and what are the outcomes of their biochemical communication. Using the attine ant fungus garden as example, samples from insect fungiculture environment could compose the initial inoculum for degradation assays, performed through interconnected **(A)** culture-dependent and **(B)** culture-independent workflows. This would allow invetigating the metabolic potential of the associated microbiota without threatening insect populations. Pencil drawing illustrations by Mariana Barcoto. PCA and heatmap were set up using random annotations, and do not comprise actual data.

To characterize the taxa composition and metabolic pathways *via* culture-dependent and –independent techniques (omics) could be an initial step to understand how the microbial community adapt to recalcitrant substrates. Part of the known microbial community found in the insect fungiculture were described in previous items, though the metabolic role and flexibility of these microorganisms are often poorly understood. For instance, besides the fungal cultivar, the core microbiota of macrotermitine termites, attine ants, bark and ambrosia beetles is composed mainly by Gammaproteobacteria, dominated by the genera *Pseudomonas, Pantoea, Klebsiella, Enterobacter*, and *Serratia.* Bacterial diversity predicted from metagenome sequences is lower than other herbivorous insects, being hypothesized to be functionally related with nutrient cycling, biofilm formation, plant degradation and detoxification, although the significance of these pathways are yet to be verified (Aylward et al., 2014; Barcoto et al., 2020; Francoeur et al., 2020). Of interest for bioremediation, the microbiota abundantly encode for genes participating in pathways for degradation of polycyclic aromatic hydrocarbons, xylene, benzoate and fluorobenzoate, ethylbenzene, styrene, cytochrome P450, chloroalkane and chloroalkene, dioxin, and naphthalene (Kirk et al., 1992; Noman et al., 2019; Barcoto et al., 2020; Chukwuma et al., 2020; Liu et al., 2021; Dhagat and Jujjavarapu, 2022). That insect fungiculture is often associated with *Pseudomonas*-enriched communities is noteworthy, since this biofilm-forming genus is reported as degrader of several xenobiotic pollutants, as PE, PET, PP, pesticides, petroleum-derived hydrocarbons, and phenols. *Comamonas* sp., *Delftia* sp., *Stenotrophomonas* sp., and *Achromobacter* sp., also figuring in some fungiculture microbiota, have been investigated for bioremediation processes. Together with lignocellulolytic fungal cultivars, these bacterial groups could compose biodegradation networks (Mann and Wozniak, 2012; Tribedi and Sil, 2013; Wasi et al., 2013; Braña et al., 2016; Kowalczyk et al., 2016; Ebadi et al., 2017; Peixoto et al., 2017; Wilkes and Aristilde, 2017; Jacquin et al., 2019; Roager and Sonnenschein, 2019; Kučić et al., 2020; Roberts et al., 2020; Varjani et al., 2020; Gambarini et al., 2021; Lear et al., 2021; Cabral et al., 2022; Hou et al., 2022). To identify possible polymer degraders in these systems, samples from insect fungiculture environment could compose the initial inoculum for degradation assays, performed through interconnected culture-dependent and culture-independent workflows (Figure 3; Shah et al., 2008b; Viljakainen and Hug, 2021). This would allow to investigate the metabolic potential of the associated microbiota without threatening insect populations.

#### Culture-Dependent Workflow

Fragments of fungus gardens, combs, galleries, adult and larvae guts could be used as initial inoculum for microbial cultivation (Figure 3A). Because these environments are apparently not homogenous (Kellner et al., 2015), composite samples could compose the inoculum. For enriching and culturing the potential degraders, the inoculum could be incubated in minimal media containing either plant or synthetic polymer as the sole carbon source. The enrichment culture could be screened for isolating microbial degraders using clear-zone assay, where the polymer is added to the agar in the form of particles, rendering an opaque media. Microbial colonies surrounded by a clear zone suggest the capacity of that colony in metabolize the polymer as carbon source, which could be identified by marker gene/region (as 16S rDNA gene or ITS) sequencing, morphological, and physiological features (as enzyme assays). Polymer-degrading enzymes could be extracted from the degrading-microbial isolates for purification, biochemical validation and characterization. Also, total genome sequencing and annotation of polymer degrading microorganisms based on databases (reviewed in detail by Dvořák et al., 2017; Jaiswal and Shukla, 2020; Saraiva et al., 2021) could assist in predicting polymer-degrading enzymes. Predicted enzymes could be tested *via* heterologous cloning, then screening positive clones using clear-zone assay (Nishida and Tokiwa, 1993; Shah et al., 2008b; Tokiwa et al., 2009; Viljakainen and Hug, 2021; Zhu et al., 2022). Culture-dependent strategies have been extensively employed for identifying plastic degrading microorganisms and enzymes, and was the method that revealed *Is*PETase from *I. sakainesis*, cultured from PET-enriched environmental samples (Yoshida et al., 2016). Coupled with screening the microbial degrading potential, plastic degradation could be quantified by determining CO_2_ evolution, O_2_ consumption, weight loss, visual and microscopic observations (Shah et al., 2008b). Alterations in polymers microstructures and microporosity could be investigated through scanning electron microscopy (SEM), Fourier transformed infrared spectroscopy (FTIR), and nuclear magnetic resonance (NMR; Bernardinelli et al., 2015; Simmons et al., 2016; Alessi et al., 2018; Li et al., 2018). While advantageous for selecting and confirming polymer- degrading activity, culture-dependent methods is limited to the small fraction of microorganisms that grow in culture, and also for being laborious (Hug et al., 2016; Viljakainen and Hug, 2021; Zhu et al., 2022).

#### Culture-Independent Workflow

From fungicultural inoculum (i.e., fragments of fungus gardens, combs, galleries, adult and larvae guts), enrichment cultures could be established as micro- or mesocosmos containing a polymer as the sole carbon source (“culturomics,” Figure 3B; Viljakainen and Hug, 2021; Zhu et al., 2022). Quantification of plastic degradation and metaomics (metagenomics, metatranscriptomics, metaproteomics, and metabolomics) could be determined for these cultures in time-serial analysis for following microbial dynamics over polymer degradation. Through Hidden Markov Models (HMMs) and Basic Local Alignment Search Tools (BLAST), homology-based metagenomics identify putative degrading pathways or enzymes according to the similarity between query sequences and known sequences deposited in databases such as: MetaCyc and BioCyc (Caspi et al., 2016), CAZy (Cantarel et al., 2009), the University of Minnesota Biocatalysis/Biodegradation Database and Pathway Prediction System (UM-BBD/PPS; Gao et al., 2010), Biochemical Network Integrated Computational Explorer (BNICE.ch; Hatzimanikatis et al., 2005), KEGG (Kanehisa et al., 2014), Plastics Microbial Biodegradation Database (PMBD; Gan and Zhang, 2019), and Plastics-Active Enzymes Database (PAZy; Buchholz et al., 2022). Such powerful method could reveal the phylogenetic distribution of plastic degraders across the microbial tree of life, highlighting the diversity and evolution of these traits (Gambarini et al., 2021). This approach may, however, overlook pathways or enzymes not sharing enough sequence similarity, thus requiring biochemical confirmation of enzyme function. The activity of putative degrading pathways requires to be confirmed by functional metagenomic screening, where the extracted DNA is cloned for heterologous expression and functional screening. Besides metagenomics, microbial dynamics could be accompanied along several time points by time-serial metatranscriptomics, metaproteomics, and metabolomics (Viljakainen and Hug, 2021; Zhu et al., 2022). A multiomics approach was employed for biodegrading PBAT using a marine microbial consortium (Meyer-Cifuentes et al., 2020), and could be adapted for using fungiculture-derived consortia.

### Lesson 2: How and When They Interact?

Efficient and productive application of microbial communities in biotechnological processes has vastly relied on synergic (i.e., cooperative) interactions between the microbiota members, which in turn can only be comprehended in the light of ecology and evolution (Cavaliere et al., 2017). Metabolic interactions in microbial communities remain to be deeply investigated in most of insect fungiculture systems, though is plausible to assume that bacteria and fungi interact in these environments. Genomics, metagenomics, biochemical, and enzymatic assays suggest a fungal-bacterial multipartite metabolism, especially for nitrogen cycling and plant degradation (Pinto-Tomás et al., 2009; De Fine Licht et al., 2010, 2013; Suen et al., 2010; Aylward et al., 2012, 2013, 2014; Grell et al., 2013; Poulsen et al., 2014; Lewin et al., 2016; Nygaard et al., 2016; da Costa et al., 2018; Barcoto et al., 2020; Ibarra-Juarez et al., 2020). For the gut microbiota of herbivores, potential syntrophic interactions may indicate productive biotechnological applications for these consortia (Cavaliere et al., 2017; Bredon et al., 2018, 2020; Gales et al., 2018; Peng et al., 2021). For insect fungiculture, putative interactions could reveal microbial influences on insect lifestyle, as well as ways to emulate them in sustainable bioprocesses. Through syntrophy or cooperation, microbial partners combine their metabolic capacity to catabolize substrates that would not be catabolized by none of the microbes alone, allowing lignocellulose degradation into fermentable sugars (Stams and Plugge, 2009; Morris et al., 2013; Cavaliere et al., 2017; Peng et al., 2021). In attine ants, for instance, the fungiculture architecture may render a spatiotemporal delimitation for microbial enzymatic activity, setting up metabolically distinct strata as subcomponents of a bioreactor operating in tandem (Somera et al., 2015). In these hypothetical bioreactors, hemicellulose (as xylan and pectin) would be first targeted, releasing monomers and oligomers that could be further metabolized in branched pathways. Subsequently, cellulose and lignin would be degraded and/or modified, deriving value-added polysaccharides and lignin derivatives (Alessi et al., 2018; Moraes et al., 2018; Chukwuma et al., 2020, 2021). Whether microbial multitrophic interactions occur in insect fungiculture, investigating them could unveil the mechanisms to modify and control the microbiome function. This knowledge would provide tools to predict and manipulate metabolic pathways, fine-tuning their enzymatic arsenal toward the degradation of agroindustrial lignocellulosic residues and xenobiotic pollutants (Saleem and Moe, 2014; Antoniewicz, 2020). Metabolic interactions could steer the engineering of interkingdom consortia, which could be designed to optimally perform specific functions, ultimately improving the outcomes of a bioprocess (Bertrand et al., 2014; Lindemann et al., 2016; Zhang et al., 2018).

Plant and plastic polymers are complex substrates, tending to favor more diverse microbial communities sharing nutritional resources and removing toxic metabolites, ultimately resulting in “biodegradation networks (Figure 1C).” Biofilms possibly define the community architecture by setting interacting populations together, thus favoring the stablishment networks “branches.” In such networks, numerous microbial species interact *via* several compounds and metabolic pathways (Pelz et al., 1999; Pazos et al., 2003; Flemming et al., 2016; Jiao et al., 2017; Puentes-Téllez and Salles, 2018; Che and Men, 2019; Kundu et al., 2019). For instance, a carbon sharing network was established in a community growing on 4- chlorosalicylate (degradation intermediate of organic pollutants) in carbon-limited conditions. Each of the three dominant members have specific ecological roles: *Pseudomonas* sp. MT1, the most abundant population, is the only strain able to convert 4- chlorosalicylate into toxic 4-chlorocatechol, which is metabolized by *Alcaligenes* sp. MT3, protecting MT1 from intermediate toxicity. Similarly, *Pseudomonas* sp. MT1 also produces toxic protoanemonin, being protected from its toxicity because the compound is metabolized by *Pseudomonas* sp. MT4. Community stability relies on microbial division of labor, based on sharing carbon skeletons (MT1), detoxifying accumulated byproducts (MT3 and MT4), and cross-feeding (Pelz et al., 1999). Properties of biodegradation networks help to predict the fate of polymer’s byproducts, which could be useful to assist the design of artificial pathways and synthetic communities (Pazos et al., 2003; Trigo et al., 2009). Aiming at composing a polymer degrading synthetic communities, synergistic and antagonistic interactions may be predicted for the microorganisms that degraded the investigated polymer in some extent. Synergistic and antagonistic interactions could be predicted from multiomics coupled with experimental validation (Li C. et al., 2016; Saraiva et al., 2021).

Besides, metagenome-assembled genomes (MAGs) could be obtained to pinpoint the metabolic contribution of individual microbial taxa involved in a given ecosystem process (López-Mondéjar et al., 2019, 2020; Peng et al., 2021; Saraiva et al., 2021; Xie et al., 2021). Indeed, MAGs were obtained from macrotermitine combs and workers gut, diverse attine fungus gardens, and bark beetles, figuring in a comprehensive catalog of metagenomes obtained from environments throughout the planet (Nayfach et al., 2021). Microbial participation in specific reactions of a metabolic reactions could be also delimited by Stable Isotope Probing (SIP), that is based on the incorporation of ^13^C-labeled substrates. Microbes capable of metabolizing specific substrates would incorporate its ^13^C version, providing a tool to track which microorganism metabolize each polymer component at each time, even uncultivated degraders. SIP may be coupled with high-resolution imaging as Fluorescent *in situ* Hybridization (FISH) and secondary ion mass spectrometry (SIMS) to identify the flux of labeled substrates between microbes (Li et al., 2008; Pett-Ridge and Weber, 2012; Seifert et al., 2012; Chokkathukalam et al., 2014; Blaser and Conrad, 2016; Musat et al., 2016; Jiang et al., 2018; Saraiva et al., 2021). Those techniques would inform on potential synergistic interactions and cooperative metabolism, expanding the understanding of ecological processes. From these data, modeling metabolic networks could be used to infer keystone species and interaction-determined dynamics, providing a theoretical framework to design effective and process-adapted microbial synthetic consortia (Faust and Raes, 2012; Stubbendieck et al., 2016; Che and Men, 2019; Faust, 2019; Vrancken et al., 2019; Shahab et al., 2020; Nikel and de Lorenzo, 2021).

### Lesson 3: Assembling Biodegrading Consortia

Consortia may be defined as groups of two or more microbial species that co-exist and interact, with the nature of their interactions determining consortia functions and stability. Diversity and redundancy of metabolic pathways fundament consortia resistance and resilience (Che and Men, 2019). With different microbial populations performing distinct but complimentary pathways in microbial consortia, the metabolic burden of degrading recalcitrant polymers may be reduced *via* division of labor. This division could imply niche-specific colonization and spatial heterogeneity for putative microbial interactions, which would have diverse outcomes according to the resource landscape (Tsoi et al., 2018; Li H. et al., 2021). Whether the microbiota composition, metabolism, and microbial interactions are known, more reliable predictions on the ecological and evolutionary outcomes of microbial interdependencies could be made (Saleem and Moe, 2014; Cavaliere et al., 2017). To assemble a biodegrading consortia, information about microbial strains, enzymes, compounds, and reactions are required. As for networks, consortia require to be studied in a holistic manner, as the whole is more than the sum of the parts, ultimately illustrating a “suprametabolism” (Pazos et al., 2003; Bhatt et al., 2021). Comprehending in detail the microbial interactions occurring in insect fungiculture systems would provide information to infer molecular mechanisms determining community assembling, as microbial communication, exchange of nutrients and energy. Such mechanisms could expand the knowledge on adaptive traits, also facilitating the design of artificial consortia. Metaomics complemented with metabolic flux analysis (for instance, by using ^13^C-labeling) are particularly useful to characterize the community structure and metabolic pathways behind these interactions (Song et al., 2014).

In designing consortia from insect fungiculture microbial members, top-down and bottom-up approaches could be applied. Top-down strategies (from complex to simple) would employ a complex community (i.e., a whole sample of gardens, combs, and galleries) for enrichment cultures with specific functions (for instance, plastic degradation). Keystone species (either degraders, detoxifiers, cross-feeders) and metabolic networks would be identified, directing the construction of optimal consortia. On the other hand, bottom-up strategies (from simple to complex) would start by selecting from a pool of microorganisms isolated from insect fungiculture environment that potentially modify the polymer. Through screening, effective consortia composition and growth conditions would be selected, being eventually optimized by metabolic engineering. These isolates may present the suitable trait, though not sharing the environmental origin. While bottom-up strategies may be simpler and easily include synthetic biology tools, they may overlook interdependent interactions naturally occurring in environmental communities, which could be preserved in top-down strategies (Sabra et al., 2010; Zuroff and Curtis, 2012; Che and Men, 2019). Examples of microbial consortia that have been employed for plastic degradation include: *Microbacterium paraoxydans* and *P. aeruginosa* (Rajandas et al., 2012), as well as *Enterobacter* sp. bengaluru-btdsce01, *Enterobacter* sp. bengaluru-btdsce02, and *Pantoea* sp. bengaluru-btdsce03 (Skariyachan et al., 2016) involved in LDPE degradation; *B. subtilis* MZA-75 and *P. aeruginosa* MZA-85 involved in PUR degradation (Shah et al., 2016), as well as *Pseudomonas* sp. and *Bacillus* sp. related to PET degradation (Roberts et al., 2020). Plastic-degrading consortia could be assembled from communities naturally occurring in insect fungiculture ecosystem and/or metabolically engineered communities. The later involve molecular biotechnology processes as gene editing and pathway engineering (Song et al., 2014; Lindemann et al., 2016).

## Conclusion

Microbial communities associated with insect fungiculture are interesting to prospect for microbes and enzymes targeting plastic degradation. Innovative biotechnological approaches were already inspired by insect gut microbiota, branching possibilities for bioremediation research (Bredon et al., 2018; Jang and Kikuchi, 2020; Kim et al., 2020; Montazer et al., 2021; Yang et al., 2021). Insect fungiculture is associated with microbial communities that often biodegrade plant polymers. Plant and synthetic plastic polymers share, in some extent, biochemical features rendering both susceptible to enzymes as peroxidases and oxidoreductases. Although highly recalcitrant, some plastic polymers are deteriorated and even partially degraded by microorganisms. By reviewing the literature on plastic polymers chemical composition, microbial degraders and degradation pathways, we found some overlapping features in plastic degrading microbiota and those associated with insect fungiculture systems. These were also reviewed to highlight their ecological importance and the promising potential of their associated microbial symbionts. Thereto, taxa composition, metabolic capacity, and microbial interactions need to be extensively comprehended. A thorough identification and characterization of microbial members and their functional potential would include a spatiotemporal comprehensive sampling, aiming at determining community oscillations and adaptive mechanisms applicable to biotechnology. This approach could unveil possible strategies for functional redundancy, then providing tools for manipulating these patterns toward specific processes, as plastics biodegradation (Lindemann et al., 2016; Dvořák et al., 2017; Antoniewicz, 2020; Jaiswal and Shukla, 2020; Ozbayram et al., 2020; Saraiva et al., 2021).

## Data Availability Statement

The original contributions presented in the study are included in the article, further inquiries can be directed to the corresponding author/s.

## Author Contributions

MOB conceived the idea, reviewed the literature, set up the figures, and wrote the manuscript. AR discussed, revised, and wrote the manuscript. Both authors contributed to the article and approved the submitted version.

## Conflict of Interest

The authors declare that the research was conducted in the absence of any commercial or financial relationships that could be construed as a potential conflict of interest.

## Publisher’s Note

All claims expressed in this article are solely those of the authors and do not necessarily represent those of their affiliated organizations, or those of the publisher, the editors and the reviewers. Any product that may be evaluated in this article, or claim that may be made by its manufacturer, is not guaranteed or endorsed by the publisher.
